# Functional Assessment of Orphan Proteins in the *Streptomyces* Pan-Proteome Through Genome-Wide Synteny Analysis

**DOI:** 10.3390/microorganisms14040791

**Published:** 2026-03-31

**Authors:** Matteo Calcagnile, Riccardo Conte, Pietro Alifano

**Affiliations:** 1 Department of Biological and Environmental Sciences and Technologies (DiSTeBA), Campus Ecotekne, University of Salento, 73100 Lecce, Italy; riccardoconte.rc@gmail.com; 2 Department of Experimental Medicine (DiMeS), Campus Ecotekne, University of Salento, 73100 Lecce, Italy; pietro.alifano@unisalento.it

**Keywords:** orphan protein, pangenome, synteny, secondary metabolism, morphological development

## Abstract

Members of the genus *Streptomyces* possess large genomes, a vast and largely unexplored metabolic potential, and a distinctive life cycle characterized by pronounced morphological differentiation. However, despite extensive molecular, genomic, and microbiological research, the functions of many genes in this genus remain poorly characterized. In this study, 929 complete *Streptomyces* genomes were analyzed. From the predicted proteomes of these genomes, proteins conserved in at least 75% of strains and lacking annotation in the KEGG GENES database were identified and clustered. To expand the annotation, synteny and co-occurrence analyses were performed between these unannotated proteins and annotated genes. A total of 330 conserved clusters were identified; 284 out of 330 clusters contain proteins encoded by genes that are syntenic with those associated with transcriptional regulation, fatty acid metabolism, two-component signaling systems, and morphological development. Additional clusters included metalloproteins and enzymes such as dehydrogenases, suggesting a wide functional spectrum. The conserved yet uncharacterized proteins identified in this analysis represent promising targets for future research, both for elucidating the molecular biology of *Streptomyces* and for expanding the range of secondary metabolites produced by these ecologically and industrially significant microorganisms

## 1. Introduction

*Streptomyces* are Gram-positive bacteria renowned for their remarkable capacity to produce a wide variety of secondary metabolites, many of which have industrial and clinical applications as antibiotics and anticancer agents [1].

The model species, *Streptomyces coelicolor*, primarily produces the antibiotics actinorhodin and undecylprodigiosin, although its genome harbors several additional biosynthetic gene clusters (BGCs) responsible for the potential production of other secondary metabolites [2,3,4]. Another well-studied member of this genus is *Streptomyces ambofaciens*, an industrial producer of spiramycin, an antibiotic used for the treatment of toxoplasmosis [5], that also synthesizes other secondary metabolites such as congocidins (netropsins) and antimycins [6,7]. Recently, spiramycin has also been shown to disarm *Pseudomonas aeruginosa* without inhibiting growth, suggesting a potential application as an antivirulence agent [8]. Numerous antibiotics are commercially produced via fermentation using *Streptomyces* species—for example, streptomycin from *Streptomyces griseus*, the first aminoglycoside antibiotic discovered, effective against *Mycobacterium tuberculosis* and various Gram-negative bacteria [9,10], and clavulanic acid from *Streptomyces clavuligerus*, a β-lactamase inhibitor commonly combined with penicillins (e.g., amoxicillin) to combat bacterial resistance [11].

Beyond antibiotics, *Streptomyces* species also produce a variety of bioactive compounds with other clinical applications. Among them is rapamycin, a potent natural immunosuppressant derived from *Streptomyces hygroscopicus*, which prevents organ transplant rejection and other clinical conditions by inhibiting the mTOR signaling pathway [12,13,14]. Ongoing research is also exploring its potential anti-aging properties [15,16].

*Streptomyces* are also characterized by a distinctive developmental life cycle [4]. These filamentous bacteria form large colonies, exhibit hyphal growth, and undergo pronounced morphological differentiation during their life cycle [17,18,19,20]. The vegetative mycelium first develops and colonizes the substrate, followed by the formation of aerial mycelium. Subsequently, the aerial hyphae undergo septation, leading to the production of exospores [17,21]. The complexity of this life cycle makes *Streptomyces* an excellent model for studying bacterial morphogenesis and the regulation of morphological development.

In addition to their complex life cycle and metabolism, *Streptomyces* are also distinguished by the remarkable complexity of their genomes [22]. These microorganisms possess large genomes, organized as linear chromosomes [22,23,24]. Typically, the *Streptomyces* genome is organized into a central “core” region containing essential genes and two chromosomal arms enriched in accessory genes that confer conditional adaptive advantages [22]. The chromosomes of widely used laboratory strains of *S. coelicolor* A3(2) contain 1.06-Mb inverted repeat sequences at their termini—known as long terminal inverted repeats (L-TIRs)—whose lengths can vary, particularly when the bacterium is cultured under laboratory conditions or exposed to random mutagenesis treatments such as UV irradiation [25]. Indeed, *Streptomyces* are well known for their remarkable genomic plasticity and can be readily manipulated in the laboratory, particularly when targeting genes located in non-core regions [22,23,26,27]. *Streptomyces* genomes are large and contain a substantial number of genes encoding orphan proteins. For instance, in *S. coelicolor*, it is estimated that approximately 34% of genes are of unknown function [28]. Therefore, one of the main objectives is to establish priority lists to guide research efforts toward the most functionally relevant proteins [28].

Since the discovery of *Streptomyces*, numerous evolutionarily conserved proteins have been identified in the *Streptomyces* genome, some of which remain functionally uncharacterized. A well-known example is WhiA, originally described as a conserved gene and later partially characterized as being essential for aerial growth, cell division, and chromosome segregation [29,30]. Given the genomic complexity of *Streptomyces* and the large number of fully sequenced genomes now available, many proteins of unknown function, called orphan proteins, can still be identified within their genomes.

Understanding the function of these orphan proteins, particularly those that are evolutionarily conserved, is of fundamental importance for both basic and applied research. Indeed, evolutionary conservation often implies that the molecular processes in which these proteins participate are also conserved. Elucidating their functions can therefore provide valuable insights into the fundamental mechanisms that govern *Streptomyces* morphology and contribute to the remarkable metabolic versatility of these bacteria.

Pangenomic and comparative genomic analyses have previously been employed to explore the diversity of BGCs and their associated metabolites, to evaluate the conservation of metabolic pathways, and to identify bacterial species with potential applications in bioremediation [31,32,33,34]. Interestingly, as revealed by genome mining studies, *Streptomyces* species typically harbor numerous BGCs, although only a few are actively expressed under standard laboratory conditions [35,36,37,38,39]. Consequently, many of these clusters are considered cryptic, and studying these orphan proteins could contribute to the development of innovative strategies aimed at activating the expression of such cryptic BGCs. For example, a pangenome constructed from 205 *Streptomyces* genomes was analyzed to evaluate the conservation of individual metabolic pathways and to identify strains with potential for metabolic engineering [33]. This study revealed that transcription factors associated with secondary metabolism, as well as biosynthetic enzymes, are largely strain-specific, whereas the biosynthetic pathways for ectoine and terpenes are highly conserved [33]. Interestingly, most genes involved in morphological development were also found to be strongly conserved, although strain-specific variants appear to fine-tune the timing of cellular differentiation.

Comparative genomics is a powerful approach that has been successfully applied to investigate plant–bacterial symbioses, characterize specific molecular systems and metabolic pathways, and elucidate the evolutionary dynamics of pathogenic bacteria, including members of the genus *Neisseria* [40,41,42,43,44]. However, comparative genomics methods are most effective when the analyzed genes are well characterized or functionally annotated using automated systems such as COG (Clusters of Orthologous Groups of proteins) or KAAS (KEGG Automatic Annotation Server) [45,46]. KAAS provides functional annotation of genes by comparing them against the manually curated KEGG GENES database [47]. Both systems offer distinct advantages and limitations. COG annotation not only assigns an identifying code to each gene but also classifies genes into broad functional categories. In contrast, KEGG annotation has the advantage of mapping each annotated gene onto the metabolic and signaling pathways represented in the KEGG database. Nevertheless, when a gene is absent from annotation databases or is annotated too generally—for example, simply as a “transcription factor”—its precise function remains unknown, and comparative genomics alone cannot provide insights beyond confirming that the gene is evolutionarily conserved.

However, even when comparative genomics fails to provide precise information about a gene—either because it is poorly characterized or not annotated—valuable insights can still be obtained through synteny analysis. In prokaryotes, functionally related genes are often organized in proximity on the chromosome, forming conserved genomic neighborhoods. This principle has been successfully applied to infer the function of a *pirin*-like protein, PirA [48,49,50], which was initially uncharacterized in *S. ambofaciens*. Similarly, comparative genomics has been employed to functionally classify RidA-like proteins in another actinomycete, *Nonomuraea gerenzanensis* [51].

In the present study, we applied a strategy based on dynamic clustering of unannotated proteins combined with KAAS and COG annotations, using the pangenome constructed from 929 *Streptomyces* strains. This approach enabled the grouping of unannotated (orphan) proteins into clusters, after which only those containing conserved proteins were further analyzed. As discussed above, synteny can provide key insights into the potential functions of unknown proteins; therefore, for each cluster, the genomic context surrounding the corresponding genes was examined to identify KAAS-annotated genes.

It is important to note, however, that physical association does not necessarily imply functional association. Nevertheless, synteny analysis—particularly when conducted across a broad range of species—can yield valuable insights and frequently represents one of the few viable investigative strategies available. Indeed, one of the most significant challenges in annotating unknown proteins is the frequent absence of detectable homology to experimentally characterized proteins—a limitation that is particularly consequential given that many of these uncharacterized proteins are likely to fulfill fundamental biological roles [52,53]. The goal of this analysis was to identify annotated genes that co-occur with orphan protein-coding genes within conserved syntenic blocks, ultimately improving functional annotation of previously uncharacterized proteins at the genome-wide level.

## 2. Materials and Methods

### 2.1. Implementation of Panproteomic Dataset and General Calculation

The initial database for this study comprised 929 *Streptomyces* genomes downloaded using ncbi_datasets [54]. Only complete genomes assembled at the whole-chromosome level were selected, and the corresponding protein sequences were retrieved, yielding a .faa file for each genome. Using custom Python 3.12 scripts (DOI: 10.5281/zenodo.18784887), the length of all proteins (scripts 1 and 2), their amino acid residue composition (%) (script 3), and the variance of amino acid composition (%) (scripts 4 and 5) were calculated and visualized for each genome.

All individual .faa files were then merged into a single dataset, generating the initial protein collection. However, this dataset contained numerous identical proteins sharing the same accession number (ID). This collection was designated the Redundant Dataset (R-D) (dataset 1, DOI: 10.5281/zenodo.18784887). To refine it, duplicate protein sequences with identical IDs were removed, producing a Non-Redundant Dataset (NR-D) (dataset 2, DOI: 10.5281/zenodo.18784887). To preserve biologically relevant information, the frequency of each identical protein across genomes was also recorded by counting the number of genomes in which a given protein ID appeared (script 6, cutoff > 50%). The fitting of the distribution curves of the number of proteins included in the clusters was performed with PAST 4 [55].

### 2.2. KAAS Annotation and Clustering

The protein sequences in the NR-D dataset were annotated using the KAAS tool, which performs protein annotation based on the KEGG GENES database [46,47]. Specifically, the bidirectional best hit (BBH) method was employed to assign orthologs. This process produced an annotation list linking each protein ID to its corresponding KEGG orthology (KO) number, while unannotated proteins were assigned a blank value (dataset 3, annotation list, DOI: 10.5281/zenodo.18784887).

To obtain a subset containing only unannotated sequences, all annotated proteins were removed from the NR-D dataset. The resulting dataset, termed NR-U-D (non-redundant unannotated dataset) (dataset 4, DOI: 10.5281/zenodo.18784887), comprised solely unannotated protein sequences. Subsequently, the NR-U-D sequences were clustered using ALFATClust to identify groups of similar proteins [56]. Each cluster was assigned a unique sequential identifier and a corresponding list of the proteins it contained.

Clusters containing only a single sequence were excluded before assessing cluster conservation. This exclusion was necessary because a single-sequence cluster indicates that the protein is unconserved or identical across all compared *Streptomyces* genomes. In such cases, the protein has already been analyzed as part of the identical protein group, as described previously. Therefore, only multi-sequence clusters were retained for the evaluation of conserved proteins.

### 2.3. Calculation of Cluster Conservation and eggNOG Annotation

Once the clusters were obtained, they were further analyzed to determine whether they contained conserved proteins. Specifically, for each cluster, the occurrence of its constituent proteins within the .faa genome files was calculated (scripts 7 and 8). In some cases, proteins belonging to the same cluster appeared multiple times within a single genome; such repeated occurrences were included in the count (scripts 9 and 10). A cluster was considered conserved if its constituent proteins were present in more than 75% of the analyzed genomes (script 11, Filtered_cluster_list.txt).

To investigate the genomic distribution of clusters, the position of each cluster within each genome was determined (cluster_pivot.py, DOI: 10.5281/zenodo.19203439). These data were then used to generate a histogram by counting the number of clusters within successive 50 kb genomic intervals (pivot_to_binmatrix.py, DOI: 10.5281/zenodo.19203439).

After calculating the conservation level of each cluster within the genomic dataset, the conserved clusters were subjected to further analysis. A preliminary quality check was conducted using the COBALT multiple sequence alignment tool [57] to ensure cluster coherence, i.e., that each cluster contained highly homologous protein sequences. Subsequently, the protein sequences belonging to each cluster were annotated using deepNOG, producing the corresponding eggNOG annotations [58,59].

### 2.4. Synenty Calculation and Functional Analysis Using KEGG Mapper

To calculate synteny, tables were constructed containing the reference gene (corresponding to the specific cluster under examination), along with the five upstream and five downstream genes. To generate these tables, the ncbi_datasets [54] utility was used to retrieve files containing the coding DNA sequences (CDS) in .fna format. These files include information on the genomic location of each gene within the sequence headers. The retrieved data were then processed to generate a comprehensive table indicating the genomic position of each gene across all analyzed genomes (dataset 6, DOI: 10.5281/zenodo.18784887).

The coordinate tables were subsequently used to generate synteny tables that included the gene belonging to the cluster under study, along with the five upstream and five downstream genes, and the genomic coordinates of each (script 12). These synteny tables were further processed by replacing the IDs of annotated proteins with their corresponding KEGG GENES codes (scripts 13 and 14). Finally, for each cluster-specific synteny table, the occurrence of annotated genes within the corresponding genomic regions across all genomes was calculated (scripts 15, 16, and 17). A cutoff of >75% (scripts 18 and 19) was applied to consider a gene as conserved within the genomic region. Genes encoding proteins conserved in *Streptomyces* (clusters) and located within regions containing conserved KAAS-annotated genes were analyzed, while the others were excluded, resulting in a final list of 330 clusters (dataset 5, DOI: 10.5281/zenodo.18784887).

To infer the functions of the unannotated proteins represented by the clusters, the genes in each syntenic region were used to perform a functional analysis using KEGG Mapper—Reconstruct [60,61].

### 2.5. Enrichment Analyses of KEGG Pathway and eggNOG Functional Class

To perform the enrichment analysis, two lists were implemented. The first list was called the observation list. It contained all the proteins annotated in the databases implemented in this study, namely the list of syntenic genes (KAAS-annotated) and the list of cluster genes (eggNOG-annotated), depending on the type of analysis performed. The second list, called the population list, contained all proteins annotated by both the eggNOG and KAAS systems of the reference genome *S. coelicolor* A3(2).

The fold enrichment was calculated with the following formula:

(1) $$\frac{k/n}{K/N}$$

where *k* is the number of proteins in the observation list that belong to a given category (COG functional class or KEGG pathway), *n* is the total number of proteins in the observation list, *K* is the total number of proteins in that category in the population, and *N* is the total number of proteins in the population.

To calculate statistical significance, the hypergeometric *p*-value (<0.05) was used. This *p*-value estimates the probability of observing at least *k* proteins in the observation list for a given category (COG functional class or KEGG pathway) by chance. The probability was computed using the hypergeometric distribution according to the following formula:

(2) $$p-value=\;P\left(X\geq k\right)=\sum_{i=k}^{min(K,n)}\frac{\left(\begin{array}{c} k\\ i \end{array}\right)\left(\begin{array}{c} N-K\\ n-i \end{array}\right)}{\left(\begin{array}{c} N\\ n \end{array}\right)}$$

where the values of *k*, *K*, *n*, and *N* are the same as in the previous formula (1), and *X* is the random variable representing the number of successes observed in *n* draws (without replacement) from a population of size *N* containing *K* successes.

This formulation estimates the probability of obtaining *k* or more successes in *n* draws (without replacement) from a population of size *N* containing *K* successes, corresponding to the likelihood that the observed enrichment occurred by chance.

To obtain a more rigorous enrichment analysis, a second calculation was performed to better estimate the effect size, and a multiple-testing correction was applied to compute the adjusted *p*-values (enrichment_fdr.py, DOI: 10.5281/zenodo.19203439). Specifically, the odds ratio (OR) was calculated from a 2 × 2 contingency table describing the presence or absence of genes associated with each pathway within the cluster compared to the rest of the dataset. This metric provides a probability-based measure of pathway overrepresentation and complements the hypergeometric test by quantifying the magnitude of enrichment. The following formula was used to calculate the OR:

(3) $$OR=\;\frac{a\times d}{b\times c}$$

where *a = k, c = K* − *k, b = n − k*, and *d = (N − K) − b*.

Multiple-testing correction was applied to adjust the *p*-values derived from the enrichment test. Raw *p*-values were corrected using the Benjamini–Hochberg false discovery rate (FDR) procedure, applied independently to each cluster. Pathways with an FDR < 0.05 were considered significantly enriched.

The Benjamini–Hochberg false discovery rate (FDR) was computed as

(4) $$q_{\left(i\right)}=\underset{\;\;\;\;\;\;\;\;j\geq i}{\min}\left(\frac{p_{\left(j\right)}\cdot m}{j}\right)$$

where $p_{\left(j\right)}$ are the ordered raw *p*-values and *m* is the total number of tests. Pathways with q<0.05 were considered significantly enriched.

### 2.6. Validation of Results

To validate the results, several approaches were employed. The first approach consisted of validating the content of each cluster using COBALT (Constraint-based Multiple Alignment Tool), as previously described [57]. In the second approach, selected conserved clusters were manually examined to confirm gene synteny. Finally, a well-known case from the literature was used as a benchmark: the syntenic proteins of the *pirA* gene in *S. ambofaciens* [48,49]. In this last case, protein sequences were extracted from the two clusters containing pirin-like genes. Since these clusters included only conserved proteins of unknown function, the *S. ambofaciens* pirin-like sequences (WP_053134172.1, WP_053127613.1, WP_053140539.1, WP_053142372.1) were used to identify additional pirin-like genes across the *Streptomyces* panproteome. This search yielded a total of 2116 pirin-like sequences.

The resulting proteins were subsequently classified using the ESM-2 (Evolutionary Scale Modeling) deep learning protein language model [62]. The resulting embedding matrix was analyzed, clustered, and visualized using the HDBSCAN (Hierarchical density-based clustering) and t-SNE (t-distributed Stochastic Neighbor Embedding) methods implemented in Python (libraries scikit-learn, matplotlib.pyplot and numpy) [63,64]. The conservation of these proteins across all genomes, as well as the corresponding synteny analyses, was performed as described previously for the genes encoding proteins included in the clusters.

In addition, the genomes of *S. ambofaciens* ATCC 23877, *S. coelicolor* A3(2), and *Streptomyces venezuelae* ATCC 10712 were manually inspected, and their genetic maps were visualized using pyGenomeViz (https://github.com/moshi4/pyGenomeViz, accessed on 20 March 2026.).

Cluster conservation and synteny were evaluated in four additional actinomycetes: *Kitasatospora setae* KM-6054 (NC_016109.1), *Micromonospora aurantiaca* ATCC 27029 (NC_014391.1), *Nocardiopsis gilva* YIM 90087 (NZ_CP022753.1), and *Frankia alni* ACN14a (NC_008278.1).

Phylogenetic relationships were inferred based on the 16S rRNA gene (~1550 bp; positions corresponding to primers 27F–1492R). Sequences were aligned using MAFFT v7 [65]. Phylogenetic reconstruction was performed with FastTree v2.1 [66] under the General Time Reversible (GTR). The phylogenetic tree was visualized using iTOL [67].

Whole-genome protein sequences for each organism were retrieved and annotated using the KAAS server, as described above. Proteins belonging to the 284 clusters identified in syntenic regions were used as queries for BLAST (blastp 2.16.0) analysis (blast_clusters.py; DOI: 10.5281/zenodo.19203439), and only hits with coverage >70% were retained (filter_blast_results.py, DOI: 10.5281/zenodo.19203439). Conserved proteins were defined based on sequence identity >70%. Synteny was subsequently assessed using genes annotated through the KAAS pipeline (synteny_check.py, DOI: 10.5281/zenodo.19203439).

## 3. Results

### 3.1. Characterization of the Dataset

**Figure 1:**
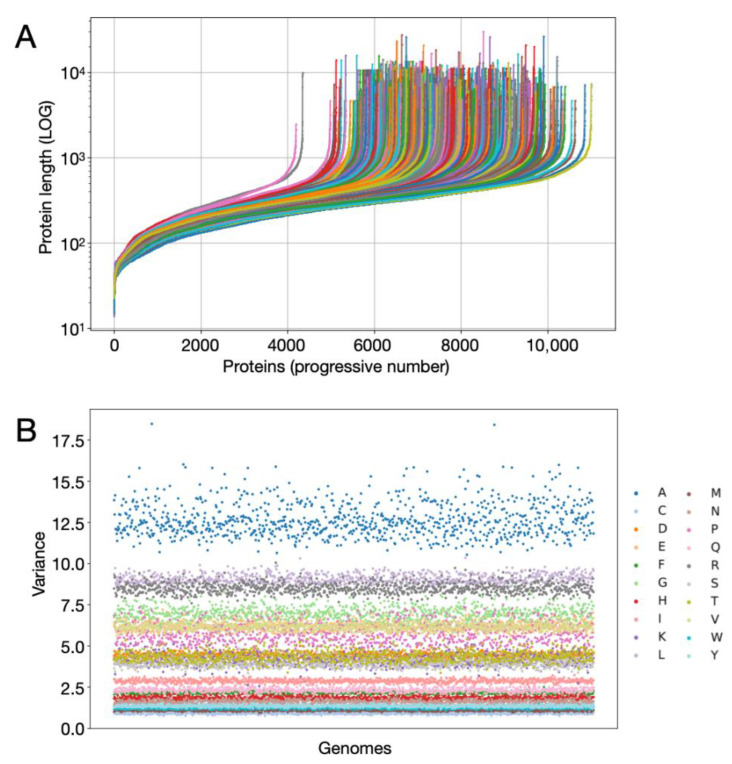
Overview of dataset features. (**A**) Protein length calculated for each genome included in the dataset. The Y-axis represents protein length, while the X-axis indicates individual proteins, each assigned a progressive identifier (at each protein was assigned a progressive identifier from longest to shortest proteins). (**B**) Variance in amino acid composition (relative abundance, %) calculated across all proteins in each genome included in the dataset.

The analyzed panproteome included protein sequences annotated in 929 complete reference genomes belonging to the genus *Streptomyces* (Table S1). The total number of protein sequences was 6,939,493. This dataset was referred to as the redundant dataset (R-D) because include identical proteins. The main characteristics of the implemented dataset are summarized in Table 1. The maximum number of coding sequences (CDS) in one genome was 11014 (*Streptomyces* sp. NBC_01450) while the minimum was 4196 (*Streptomyces* sp. MRC013) with an average of 7469.85 CDS per genome (Table 1 and Table S2). The length of the predicted protein sequences ranged from 30019 amino acids (*Streptomyces* sp. NBC_01717) to 14 amino acids (*S. europaeiscabiei* NBC_00494, *S. europaeiscabiei* NBC_00480, *Streptomyces* sp. R08) (Figure 1A, Table 1 and Table S2).

Protein sequences contained in each genome of this dataset were analyzed globally by calculating the amino acid composition of each protein (%) and, subsequently, calculating the variance of the % of each amino acid in each genome (Figure 1B, Table S2). As can be seen, alanine (A) is the amino acid with the greatest variance (range 18.48–10.54), followed by leucine (L, range 10.35–8.26), arginine (R, range 10.06–7.59), glycine (G, range 8.95–6.14) and proline (P, range 8.00–4.66). A series of amino acids have an intermediate variance: valine (V, range 7.24–5.53); glutamic acid (E, range 7.20–5.49); Aspartic Acid (D, range 5.01–4.04); lysine (K, range 5.34–2.55); threonine (T, range 5.50–3.36) and serine (S, range 4.93–3.35). Furthermore, 5 amino acids have a low variance range, ranging from values greater than 1 (>1) to values greater than 3 (>3): I, range 3.23–2.60; Q, range 3.00–2.02; F, range 2.50–1.97: H, range 2.28–1.55; N, range 2.84–1.29. Finally, 4 amino acids have low variance values: tyrosine (Y, range 1.62–1.17); tryptophan (W, range 1.46–1.08), methionine (M, range 1.36–0.94), and cysteine (C, range 1.39–0.76).

### 3.2. Spreading of Non-Redundant (Identical) Proteins

The protein sequence dataset was implemented using protein sequences predicted from the complete genomes. This means that proteins with the same ID could be present in each genome, as described previously. This was true for 928 of the 929 analyzed genomes because the proteins were classified using a different type of ID for the *S. coelicolor* GCF_000203835.1. Therefore, the GCF_000203835.1 proteome was analyzed manually.

Identical proteins—excluded from the non-redundant dataset for computational efficiency—were analyzed separately, as their inclusion in the clustering pipeline would have generated uninformative clusters of identical sequences. Notably, the majority of these proteins correspond to highly conserved and well-characterized components ubiquitous across *Streptomyces* genomes, including ribosomal proteins, whose functions are already well established in the literature.

**Figure 2:**
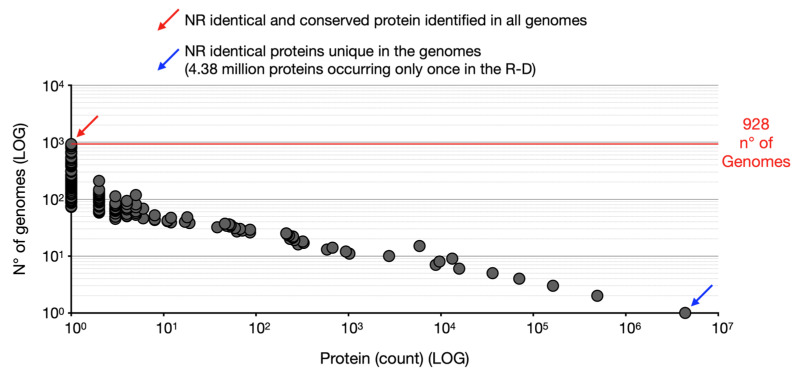
Distribution of identical proteins across analyzed genomes. The Y-axis represents the number of genomes in which each protein was identified, while the X-axis indicates the number of proteins corresponding to each occurrence value among the genomes.

Of the 6,939,493 protein sequences contained in the R-D (initial dataset), 5,211,181 unique IDs were identified and were therefore included in the non-redundant dataset (NR-D). The number of identical proteins from NR-D occurring at least once in the R-D has been calculated and plotted in Figure 2. Most of the data is concentrated in the lower values, with more than 4 million proteins (4,387,197) occurring only once in the R-D, and almost 500 thousand proteins occurring twice in the R-D, about 160 thousand proteins occurring three times in the R-D, about 70 thousand proteins occurring four times in the R-D, and about 36 thousand proteins occurring five times in the R-D (Figure 2). The data suggest a highly skewed distribution, with a long tail of low-frequency values.

Only 11 proteins were present in at least 50% of the analyzed genomes (464) and are reported in Table 2.

As expected, 6 ribosomal proteins were present in this group of identical proteins: 30S ribosomal protein bS22 (WP_003948845.1, present in 99.89% of the genomes); 30S ribosomal protein S10 (WP_003948644.1, 92.68%); 30S ribosomal protein S12 (WP_003948652.1, 84.28%); type Z 30S ribosomal protein S14 (WP_003948630.1, 58.56%); 30S ribosomal protein S11 (WP_003956432.1, 55.65%) and 50S ribosomal protein L36 (WP_003956441.1, 52.42%) (Table 2). In addition to these proteins, two proteins involved in morphological differentiation in Streptomyces were present: BldC (WP_003949541.1, 91.07%) and SsgA (WP_003959770.1, 52.31%). BldC is a short peptide that belongs to the MerR family [68]. It is a transcription involved in regulating the development and antibiotic production in *Streptomyces* [68], while SsgA is a regulator involved in sporulation and cell division [69]. Another protein that is highly conserved in the pangenome of *Streptomyces* is the transcription factor CarD (WP_003953493.1, 68.78%) that is essential in *Mycobacterium tuberculosis* because it stabilizes the RNAP holoenzyme/DNA complex [70,71]. Another highly conserved protein, WP_003948568.1, was found to be identical in 98.28% of the analyzed genomes. This protein belongs to the RpfG family and contains an HD-GYP domain, which is involved in the degradation of cyclic di-GMP. It functions as part of a two-component signal transduction system that regulates virulence in *Xanthomonas campestris* pv. *Campestris* [72]. Finally, a less conserved protein, present in 72.98% of the analyzed genomes, is WP_003966491.1, which contains a DUF3117 domain of unknown function. Proteins containing the DUF3117 domain constitute a family of small, uncharacterized bacterial proteins (typically 50–60 amino acids in length) identified in various actinobacteria, including *Kineococcus radiotolerans*, *Arthrobacter*, and *Mycobacterium*. Studies on antibiotic responses in mycobacteria have shown that DUF3117-domain-containing proteins exhibit altered translation dynamics under pharmacological treatments; however, their precise biological role remains unknown [73].

### 3.3. Clustering of Unannotated Protein

As previously described, 5,203,029 protein sequences were included in the NR-D and were associated with a unique ID. These proteins were annotated using KAAS, resulting in a list of 1,843,418 annotated proteins. The annotation list was used to annotate all 929 genomes in the dataset using the KEGG classification system. The genomes were then parsed to obtain a non-redundant unannotated dataset (NR-U-D) containing 3,547,624 unannotated proteins belong to the *Streptomyces* panproteome.

**Figure 3:**
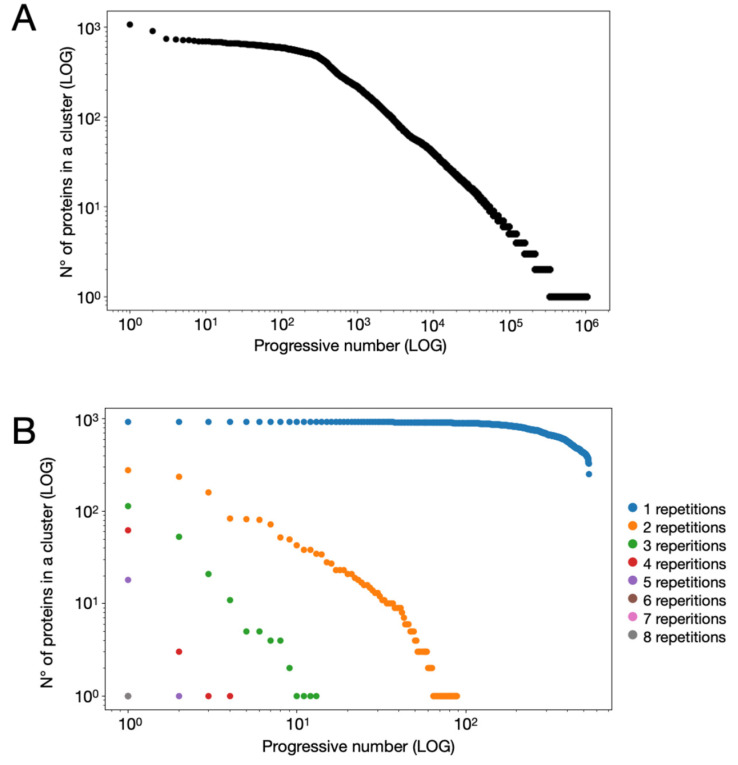
Distribution of proteins belonging to each cluster. The Y-axis represents the number of proteins per cluster, while the X-axis corresponds to the cluster order (at each cluster was assigned a progressive identifier from the cluster that includes more proteins to the cluster that includes a lower number of protein). (**A**) Comprehensive distribution of proteins across all clusters. (**B**) Distribution of proteins within each cluster, sorted according to the frequency of protein repetitions within individual genomes across the entire genome database.

ALFATClust [56] was used to cluster the proteins contained in the NR-U-D dataset. The COBALT multiple alignment tool was used to validate the content of each cluster [57]. However, due to the large number of clusters obtained, this validation was performed only after identifying the phylogenetically conserved clusters, as described in the following section. This process resulted in 1,043,336 clusters, of which 704,127 contained a single sequence (singleton cluster). The dataset containing the clusters was named C-NR-D (clustered non-redundant dataset). The number of proteins belonging to each cluster was used to generate a distribution graph in which the number of proteins per cluster is represented on the x-axis while the y-axis represents the order of the clusters sorted from the one containing more proteins to the one containing less proteins (Figure 3A). As can be seen, the curve has a trend characterized by two inflection points: initially (more numerous clusters) there is a rapid decrease in the curve followed by a plateau phase and a further rapid decrease. To characterize the curve, clusters containing equal numbers of proteins were considered only once, and the curve was analyzed using the nonlinear fitting models of PAST software (Figure S1A). According to this analysis, the best models for the curve are the exponential model (increasing decay) (R^2^ = 0.9913) and the sigmoid Hill’s curve (R^2^ = 0.9929) (Figure S1B,C).

Subsequently, the clusters derived from unannotated proteins were analyzed by examining the repetitions of each protein within each genome across the entire genome database. The resulting distribution is shown in Figure 3B, where the x-axis represents a numeric alias obtained by ordering the clusters according to the number of proteins contained in each cluster. It can be observed that proteins belonging to clusters with single occurrences are the most abundant and dominate the upper part of the graph, indicating very large clusters. Proteins repeated twice display a decreasing distribution, showing a clear trend toward progressively smaller cluster sizes. Proteins with higher repetition numbers (from three to eight) are increasingly fewer.

### 3.4. Cluster Conservation in the 929 Genomes

After analyzing the clusters and repeat IDs in each genome, we assessed the conservation of each cluster. Clusters whose member proteins were present in at least 75% of the analyzed genomes were defined as conserved clusters. This analysis revealed that 330 clusters were localized in conserved genomic regions, including Cluster 3, which contains annotated proteins of the BldC family. As previously noted, many streptomycete genomes included in this study contain an identical BldC protein (WP_003949541.1, Table 2), and the other protein IDs in Cluster 3 also share the same sequence. As mentioned above, the content of each cluster was validated using COBALT multialignment tool [57], confirming that the protein sequences within each cluster were uniform and consisted of homologous sequences. Of these, 205 clusters had proteins present only once per genome (Table S3), 40 clusters were duplicated in a single strain (Table S4), and 30 clusters were duplicated in two or more strains (Table S5). Among the latter, Cluster 14531 exhibited the highest degree of duplication, occurring in 76 strains, followed by Cluster 9943 (28 strains), Cluster 2065 (25 strains), Cluster 1226 (19 strains), and Cluster 14352 (11 strains). Two clusters, Cluster 14026 and Cluster 19662, were duplicated in 9 strains each, while Cluster 34477 was duplicated in 7 strains. Cluster 19775 and Cluster 13703 were duplicated in 6 strains, and Cluster 19445 was duplicated in 5 strains. Five clusters—Cluster 14180, Cluster 14508, Cluster 50, Cluster 16729, and Cluster 15088—were duplicated in 3 strains, and 14 clusters—Cluster 3509, Cluster 3682, Cluster 6798, Cluster 14374, Cluster 14532, Cluster 14538, Cluster 14573, Cluster 14616, Cluster 16548, Cluster 24325, Cluster 32590, Cluster 32591, Cluster 13958, and Cluster 13909—were duplicated in 2 strains.

Several clusters exhibited both duplication and triplication across the analyzed genomes (Table S6). Cluster 13 was duplicated in 1 strain and triplicated in 5 strains, while Cluster 41 was duplicated in 37 strains and triplicated in 2 strains. Cluster 10414 was duplicated in 53 strains and triplicated in 4 strains, and Cluster 48615 was duplicated in 88 strains and triplicated in 11 strains. Cluster 19209 was duplicated in 2 strains and triplicated in 1 strain, Cluster 16417 was duplicated in 28 strains and triplicated in 10 strains, and Cluster 15756 was duplicated in 10 strains and triplicated in 1 strain.

Some clusters exhibited higher-order duplications across the analyzed genomes (Table S6). Cluster 15448 was duplicated in 43 strains and quadruplicated in 1 strain. Cluster 13576 showed extensive variation, being duplicated in 248 strains, triplicated in 75 strains, quadruplicated in 10 strains, and quintuplicated in 2 strains. A singular case is Cluster 14144 (Table S7), which exhibits remarkable variation in copy number across the analyzed genomes. It is present as a single copy in 340 strains, duplicated in 234 strains, triplicated in 120 strains, quadruplicated in 59 strains, quintuplicated in 21 strains, and present in six copies in 11 strains. Rarely, it is found in seven copies in 2 strains, eight copies in 3 strains, and eleven copies in 1 strain. The remaining clusters not discussed in this paragraph are either not located in syntenic regions or the syntenic regions do not contain genes annotated with the KAAS annotation tool.

**Figure 4:**
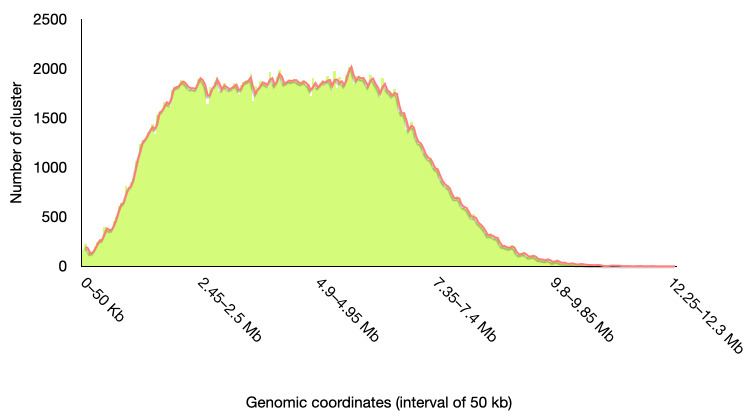
Genomic coordinates of clusters calculated in the 929 genomes, with 50 Kb intervals. Clusters that include conserved proteins and that are located in conserved genomic regions (284 of 330) were analyzed.

As described below, 284 of the 330 clusters are located in conserved genomic regions that include the cluster protein along with other syntenic elements. *Streptomyces* species possess a plastic genome organized into two distinct components: a central “core” region comprising conserved genes, and two terminal arms enriched in less conserved and more variable genes. Notably, the genomic positions of these 284 clusters are consistent with this architectural organization (Figure 4). Specifically, the number of conserved proteins within the clusters is highest in the central region of the genome and decreases toward both chromosomal arms (Figure 4).

Indeed, when focusing the analysis on single clusters containing non-duplicated proteins in *Streptomyces*, we observe a similar pattern of conservation (Figure S1A, Table S8). In contrast, when examining clusters that include proteins present in multiple copies across the analyzed genomes, the extent of conserved proteins is reduced (Figure S1B, Tables S9 and S10).

### 3.5. Genome-Wide Synteny Reveals Orphan Proteins with Conserved Genomic Neighborhood in Streptomyces

As described in the introduction, the primary aim of this study was to predict the cellular functions of conserved yet uncharacterized proteins in *Streptomyces* using a syntenic approach. To achieve this, we analyzed synteny at the pangenomic level. Genomic maps were constructed for each genome and for each cluster, including the coding gene of interest along with the 5 upstream and 5 downstream genes (neighborhood genes), for a total of 11 genes per map. After constructing the maps, each protein ID was replaced with the corresponding code from the previously calculated KAAS annotation. Finally, the co-occurrence of genes from the cutlers with KAAS-annotated neighborhood genes was calculated (Tables S8–S10). To identify which functional categories were most closely associated with genes neighboring those in the clusters, we used the *S. coelicolor* genome as a reference to calculate enrichment.

**Figure 5:**
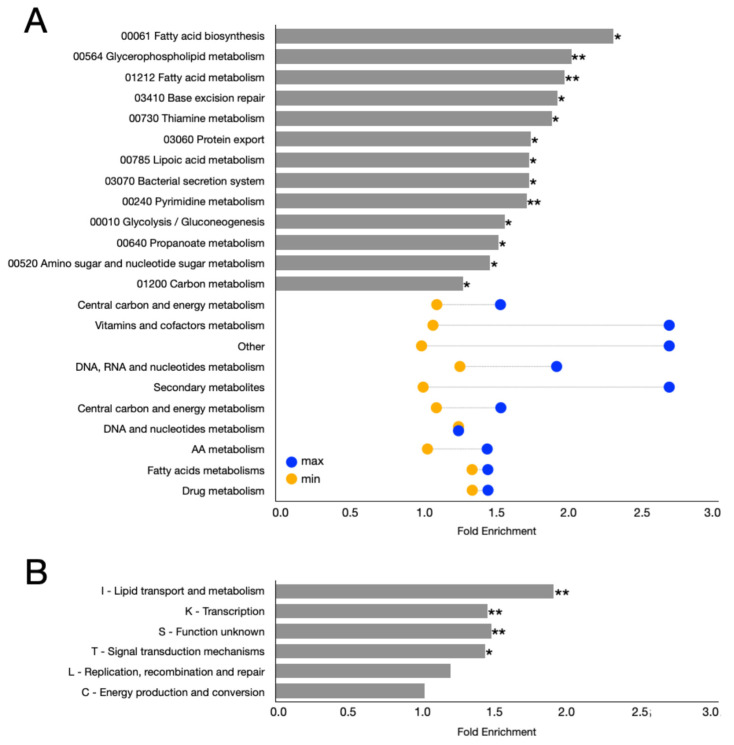
Enrichment analyses. (**A**) Enrichment of KEGG pathways calculated for genes syntenic to those included in the clusters. (**B**) Enrichment of COG categories assigned to each cluster. * = statistically significant enrichment (*p*-value < 0.05); ** = statistically significant enrichment (*p*-value < 0.01).

Among the most enriched categories, 14 KEGG pathways showed statistically significant enrichment with *p*-value < 0.05 (Figure 5A). These included: Basal transcription factors (03022), Pyrimidine metabolism (00240), Fatty acid metabolism (01212), Glycerophospholipid metabolism (00564), Base excision repair (03410), Fatty acid biosynthesis (00061), Glycolysis/Gluconeogenesis (00010), Protein export (03060), Amino sugar and nucleotide sugar metabolism (00520), Carbon metabolism (01200), Lipoic acid metabolism (00785), Bacterial secretion system (03070), Thiamine metabolism (00730), and Propanoate metabolism (00640) (Figure 5A). However, the Basal transcription factors category (03022) was represented by only a single gene among the syntenic genes associated with the clusters and was absent from the reference genome. Therefore, this category was excluded from the analysis.

Interestingly, several pathways involved in secondary metabolism (00997, 00999, 00524, 00520, and 01110) were not enriched (Figure 5A), nor was the Prodigiosin biosynthesis pathway (00333) (Figure 5A). However, it is important to emphasize that many of the genes included in the identified clusters, as well as numerous genes syntenic to them, show direct or indirect connections to secondary metabolism and morphological differentiation in *Streptomyces*.

Other pathways related to central metabolism—such as Pyruvate metabolism (00620), the Pentose phosphate pathway (00030), Fructose and mannose metabolism (00051), and the Citrate cycle (TCA cycle) (00020)—were also not enriched, in contrast to Glycolysis/Gluconeogenesis (00010) and Carbon metabolism (01200), which were enriched (Figure 5A). Similarly, while the Fatty acid biosynthesis pathway (00061), and Fatty acid metabolism (01212) were enriched, the Fatty acid degradation pathway (00071) was not. (Figure 5A) Indeed, there is a well-established link between fatty acid metabolism and secondary metabolism in bacteria [25,48,49]. In fact, the molecular machinery involved in polyketide synthesis—comprising the enzymes known as polyketide synthases (PKSs)—is highly similar to that responsible for fatty acid synthesis (FAS, fatty acid synthase).

It is also noteworthy that several amino acid metabolic pathways, including C5-Branched Dibasic Acid metabolism (00660), Valine, Leucine, and Isoleucine degradation (00280), Lysine biosynthesis (00300), Alanine, Aspartate, and Glutamate metabolism (00250), Cysteine and Methionine metabolism (00270), Histidine metabolism (00340), and Valine, Leucine, and Isoleucine biosynthesis (00290), were not enriched (Figure 5A). Likewise, pathways associated with nucleotide and metabolism—such as Nucleotide metabolism (01232) and Biosynthesis of nucleotide sugars (01250)—were not enriched (Figure 5A), in contrast to Pyrimidine metabolism (00240), and Amino sugar and nucleotide sugar metabolism (00520), which were significantly enriched (Figure 5A). Pathways related to RNA synthesis, such as RNA degradation (03018), Aminoacyl-tRNA biosynthesis (00970), and RNA polymerase (03020), were also not enriched (Figure 5A).

Although the initial analysis revealed a statistically significant correlation between some pathways associated with syntenic proteins and conserved clusters, this correlation should be interpreted as indicative of a potential functional link rather than definitive evidence. To strengthen these findings, a multiple-testing correction was applied to adjust the *p*-values derived from the enrichment test. Raw *p*-values were corrected using the Benjamini–Hochberg false discovery rate (FDR) procedure.

When this correction was applied to the enrichment analysis performed on all annotated proteins (i.e., aggregating all clusters), the fold enrichment (FE) of most pathways was no longer statistically significant (adjusted *p*-value > 0.05) (Table S11). This indicates that the observed enrichment signals do not withstand rigorous multiple-testing correction and should therefore be considered exploratory. However, four metabolic pathways remained significant, including 00909 Sesquiterpenoid and triterpenoid biosynthesis (OR = 112), 00906 Carotenoid biosynthesis (OR = 112), and 01110 Secondary metabolite biosynthesis (OR = 4.9) (Table S11). The fourth pathway (00997 Various other secondary metabolite biosynthesis) was manually excluded because it was represented by a single copy in the reference genome.

The same analysis was then performed independently for each cluster, identifying 318 enriched pathways across 151 clusters (Table S12). For example, cluster 14573 (adenosyl-hopene transferase HpnH) showed enrichment for pathway 01110 Secondary metabolite biosynthesis, and indeed all four syntenic genes in this cluster were classified within pathway 01110. A complete list of results is provided in Table S12.

Beyond these statistically significant data, these results should be interpreted as exploratory: enrichment reflects statistical over-representation of K numbers within a cluster relative to the reference background and does not constitute evidence of direct functional involvement. Results are subject to reference genome selection bias and uneven KEGG annotation coverage, particularly for pathways with sparse or lineage-specific curation.

### 3.6. EGGNOG Annotation of KAAS-Unannotated Conserved Protein

To further analyze the results obtained from the synteny and enrichment analyses described above, eggNOG annotation was performed using DeepNOG [58,59] on the protein sequences contained within the 330 previously identified clusters. An additional enrichment analysis was then carried out directly on the COG category of the proteins belonging to these clusters (Figure 5B).

This analysis revealed enrichment in four functional categories: Lipid transport and metabolism (I), Transcription (K), Function unknown (S), and Signal transduction mechanisms (T) (Figure 5B). Notably, the predominance of the Function unknown (S) category indicates that most of the identified clusters—and therefore the proteins they contain—remain uncharacterized, supporting the effectiveness of this approach in detecting previously unknown proteins. The enrichment of the Lipid transport and metabolism (I) category further suggests that many of these proteins are associated with lipid-related processes, consistent with the synteny-based enrichment observed for the Fatty acid biosynthesis (00061) and Fatty acid metabolism (01212) pathways (Figure 5A,B).

Interestingly, the Signal transduction mechanisms (T) category was enriched in this analysis, whereas the Two-component system pathway (02020) was not enriched in the KAAS-based syntenic protein analysis (Figure 5A,B). This discrepancy suggests that some cluster-associated proteins may participate in two-component systems that regulate other cellular processes or metabolic pathways. Finally, the Transcription (K) category was enriched here, while many RNA metabolism-related categories were not enriched in the previous synteny analysis (Figure 5B). This finding implies that the cluster-associated proteins are more likely linked to specific transcriptional regulators rather than to the general molecular machinery involved in RNA synthesis or degradation.

### 3.7. Clusters 3 and 11 Connect bldC and bldD with Pyrimidine and Amino Acid Metabolisms

Cluster 3 contains sequences encoding a regulatory factor associated with the *bldC* regulator. This locus lies within a conserved syntenic block that includes *hrpA* (ATP-dependent RNA helicase; K03578), *purF* (amidophosphoribosyltransferase; K00764), *purM* (phosphoribosylformylglycinamidine cyclo-ligase; K01933), and *vdh* (valine dehydrogenase [NAD^+^]; K00271) (Table S8). By contrast, cluster 11 contains proteins annotated as the transcriptional regulator BldD, which is encoded in a different conserved region (Table S8). That second syntenic neighborhood includes *nusB* (transcription antitermination protein NusB; K03625), *efp* (elongation factor P; K02356), and a contiguous set of four genes involved in pyrimidine metabolism: *carA* (K01956), *pyrC* (K01465), *pyrB* (K00609) and *pyrR* (K02825). Using the KEGG reconstruction tool KEGG Mapper, it was observed that the genes K00271, K00764, K01933, and K01956 are part of the Biosynthesis of secondary metabolites pathway (01110), whereas K00609, K01465, K01956, and K02825 belong to the Pyrimidine metabolism pathway (00240), specifically participating in the conversion of amino acids into other intermediates of primary and secondary metabolism. These findings suggest a potential connection between the regulation of secondary metabolism, pyrimidine metabolism, and amino acid metabolism.

### 3.8. Several Conserved Clusters Are Associated with Transcription Regulation and Transcription Machinery

After completing the enrichment analyses, a specific cluster-level analysis was performed. This initial analysis focused on clusters containing proteins encoded by non-redundant genes within the *Streptomyces* genome. Of the 205 clusters containing non-redundant genes (Table S8), the majority—44 clusters—include genes annotated as transcription factors belonging to different families, including the IclR (4 clusters), Lrp/AsnC (3 clusters), MarR (3 clusters), TetR (6 clusters) and GntR (4 clusters) families.

To determine whether these transcription factors could be correlated with specific metabolic or regulatory functions in *Streptomyces*, a KEGG Mapper analysis was performed separately for each family of transcriptional regulators. Overall, all transcription factors were associated with metabolic functions. Specifically, members of the IclR family were linked to Biosynthesis of secondary metabolites (01110); the Lrp/AsnC family to Biosynthesis of cofactors (01240), particularly thiamine metabolism; the MarR family to Biosynthesis of nucleotide sugars (01250), Galactose metabolism (00052), and Amino sugar and nucleotide sugar metabolism (00520); the TetR family to Microbial metabolism in diverse environments (01120) and Carbon metabolism (01200); and the GntR family to Biosynthesis of secondary metabolites (01110), Microbial metabolism in diverse environments (01120), Carbon metabolism (01200), 2-Oxocarboxylic acid metabolism (01210), Glycolysis/Gluconeogenesis (00010), Citrate cycle (TCA cycle) (00020), Pyruvate metabolism (00620), and Lipoic acid metabolism (00785).

In addition to these families represented in multiple clusters, 5 transcription factors belonged to families represented by a single cluster each (Table S8). As with the other transcription factors, these were generally associated with metabolic processes. For example, CdaR (Cluster 20511) was correlated with Fatty acid metabolism (01212) and Fatty acid biosynthesis (00061); DeoR (Cluster 10226) with Biosynthesis of secondary metabolites (01110); LuxR (Cluster 15606) with Biosynthesis of nucleotide sugars (01250) and Galactose metabolism (00052); and YebC/PmpR (Cluster 19331) with several systems, including Biosynthesis of cofactors (01240), Lipoarabinomannan (LAM) biosynthesis (00571), Vitamin B6 metabolism (00750), Protein export (03060), Homologous recombination (03440), and the Bacterial secretion system (03070).

The LysR regulator (Cluster 15921) was not associated with any specific pathway but was located in a syntenic region containing an *rpoE* gene and a gene encoding a WhiB-family redox-sensing transcriptional regulator, suggesting that this LysR factor may participate in broader, pleiotropic transcriptional regulation. Other single-cluster transcription factors included MurR/RpiR (Cluster 9865), found in a syntenic region containing the *murQ* gene, which encodes an enzyme essential for bacterial cell-wall recycling and metabolism, and PadR (Cluster 16627), co-occurring with *pcnB*, a gene encoding poly(A) polymerase potentially involved in RNA degradation control in *Streptomyces*.

In addition to these transcription factors annotated as belonging to specific families, 17 others were classified under general regulatory categories (Table S8). Two of these were annotated as helix–turn–helix proteins and were found in genomic regions containing a D-Ala-D-Ala carboxypeptidase gene and an ALDH (aldehyde dehydrogenase) gene, respectively. The remaining 15 transcription factors were analyzed collectively using KEGG Mapper, revealing correlations with several pathways, including: Biosynthesis of secondary metabolites (01110), Microbial metabolism in diverse environments (01120), Biosynthesis of nucleotide sugars (01250), Biosynthesis of cofactors (01240), Galactose metabolism (00052), Oxidative phosphorylation (00190), Purine metabolism (00230), Amino sugar and nucleotide sugar metabolism (00520), ABC transporters (02010), Two-component system (02020), and Quorum sensing (02024). Among these transcription factors, Cluster 15362 is located in a genomic region containing the *mtrA* and *mtrB* genes, which encode the two components of a two-component regulatory system belonging to the OmpR family, as well as the *secA* gene, which encodes a protein involved in the secretion system. Interestingly, Cluster 15363 includes proteins annotated as ribosome hibernation-promoting factors (HPF/YfiA) and is located within the same syntenic region as *secA*, *mtrA*, and *mtrB*. Cluster 1 contains proteins annotated as response regulator transcription factors and is located in a syntenic region that includes the *rpoE* and *whiB* genes, as well as two genes encoding enzymes—UDP-*N*-acetyl-*D*-glucosamine dehydrogenase and *guaB* (IMP dehydrogenase).

In addition to transcription factors, several clusters contain proteins of unknown function that may exert a more profound influence on transcriptional regulation. Among these, two clusters—Cluster 4 and Cluster 12—include protein sequences annotated as RNA polymerase-binding protein RbpA (Table S8). The genes encoding the proteins in Cluster 4 are located in syntenic regions containing genes associated with the Biosynthesis of secondary metabolites (01110), Microbial metabolism in diverse environments (01120), Carbon metabolism (01200), Biosynthesis of amino acids (01230), and Glycolysis/Gluconeogenesis (00010) pathways. In contrast, the Cluster 12 genes are found in regions that contain genes belonging to various metabolic pathways.

Two additional clusters, Cluster 2989 and Cluster 9612, include proteins annotated as the sigma factor SigE (Table S8), which co-occur syntenically with distinct gene pairs: in the first case, *udk* (uridine kinase) and *mtrB* (a two-component system sensor histidine kinase of the OmpR family), and in the second, *disA* (diadenylate cyclase) and *mutY* (A/G-specific adenine glycosylase). Another cluster, Cluster 15722, includes proteins annotated as RNA polymerase sigma factor RpoD/SigA (Table S8), co-occurring syntenically with a gene encoding 4,5-DOPA dioxygenase extradiol.

Interestingly, three clusters—Cluster 25586, Cluster 5689, and Cluster 5696—are located in a syntenic region containing the *gyrA* gene, which encodes DNA gyrase subunit A (Table S8). Cluster 25586 includes a GntR family transcriptional regulator, whereas Clusters 5689 and 5696 contain proteins annotated as M16 family metallopeptidases (Table S8). This genomic region also encompasses the *ptsH* gene, which encodes the phosphocarrier protein HPr. Another cluster, Cluster 13751, also includes M16 family proteins, but its syntenic region contains *thyX* (thymidylate synthase), *rpsO* (ribosomal protein S15), *pnp* (polyribonucleotide nucleotidyltransferase), and *dapB* (4-hydroxy-tetrahydrodipicolinate reductase).

Even more noteworthy is Cluster 55, which contains a hypothetical protein located in a syntenic region encompassing the *gyrB* gene (encoding the B subunit of DNA gyrase) and the *rpoD* gene (encoding the RNA polymerase primary sigma factor) (Table S8).

In addition to the clusters and genes described above, it is important to note that several other clusters are located in syntenic regions containing genes involved in transcription or translation processes, such as those encoding ribosomal proteins (Cluster 2).

### 3.9. Several Conserved Clusters Are Associated with Dehydrogenase or Oxidoreductase Enzymatic Activity, and with Metalloprotein

Several clusters (Cluster 1796, Cluster 9985, Cluster 10206, Cluster 10837, Cluster 13708, Cluster 13842, Cluster 15319, Cluster 21672, and Cluster 21926) include genes annotated as various types of dehydrogenases, such as acyl-CoA dehydrogenase, aldehyde dehydrogenase, zinc-binding dehydrogenase, and xanthine dehydrogenase (Table S8). KEGG Mapper analysis revealed that these enzymatic activities are associated with multiple metabolic processes, particularly the Biosynthesis of secondary metabolites pathway (01110).

Similarly, seven clusters (Cluster 6813, Cluster 10685, Cluster 15665, Cluster 15668, Cluster 16745, Cluster 19867, and Cluster 20077) contain genes annotated as oxidoreductases (Table S8). KEGG Mapper analysis of the syntenic regions associated with these oxidoreductases indicated relationships with several pathways, including Quorum sensing (02024), Base excision repair (03410), ABC transporters (02010), and Biosynthesis of secondary metabolites (01110).

The remaining twelve clusters (Cluster 91, Cluster 5397, Cluster 9431, Cluster 9583, Cluster 10635, Cluster 11799, Cluster 19319, Cluster 19712, Cluster 19814, Cluster 20086, Cluster 20774, and Cluster 20790) (Table S8) contain genes encoding various metalloproteins, including numerous metallopeptidases, as well as proteins related to iron and sulfur metabolism—such as iron–sulfur cluster insertion protein ErpA, metal–sulfur cluster assembly factors, and radical SAM family heme chaperone HemW. Collectively, these proteins were associated with the Biosynthesis of secondary metabolites (01110) and Biosynthesis of cofactors (01240) pathways. As described previously other Clusters that include metalloproteins (M16 family peptidases) are Clusters 5689, Cluster 5696 and Cluster 13751.

### 3.10. Other Genes That Are Syntenic with Those Belonging to Conserved Clusters

In addition to the clusters already discussed, several others include genes annotated as class I SAM-dependent methyltransferases (Cluster 9477, Cluster 9731, Cluster 13962, Cluster 19854, and Cluster 20382), as NUDIX domain-containing proteins (Cluster 13856, Cluster 16686, and Cluster 19423), among others (Table S8).

Several clusters (Cluster 90, Cluster 9204, Cluster 9408, Cluster 10535, Cluster 13676, Cluster 13885, Cluster 13911, Cluster 16358, Cluster 16387, Cluster 19327, Cluster 19529, Cluster 19972, Cluster 20047, Cluster 20220, Cluster 20509, Cluster 20766, and Cluster 21473) include proteins annotated as containing the following domains of unknown function (DUFs): DUF2252, DUF2469, DUF2617, DUF3039, DUF3040, DUF3090, DUF3097, DUF3145, DUF3151, DUF349, DUF4177, DUF4191, DUF4193, DUF5926 (SEC-C domain-containing), DUF5998, DUF6758, and DUF948 (Table S8). Overall, these proteins are encoded by genes located in syntenic regions that also contain genes associated with several metabolic pathways, including Biosynthesis of secondary metabolites (01110), Microbial metabolism in diverse environments (01120), Fatty acid metabolism (01212), and Biosynthesis of cofactors (01240).

Proteins annotated as hypothetical are included in nine clusters, including the previously discussed Cluster 55, as well as Cluster 2067, Cluster 5592, Cluster 6630, Cluster 13649, Cluster 16330, Cluster 17234, Cluster 20096, and Cluster 20787 (Table S8). The genes encoding these proteins are located in syntenic regions that also contain genes associated with Microbial metabolism in diverse environments (01120), Biosynthesis of cofactors (01240), and Quorum sensing (02024).

Among the other clusters, Cluster 13896 and Cluster 13772 contain proteins involved in morphological differentiation in *Streptomyces*: the septation protein SepH and a SpoIIE family protein phosphatase, respectively (Table S8). The gene encoding the latter is located in a syntenic region that includes *spoIIIE*, *rnj* (ribonuclease J), and *rimO* (ribosomal protein S12 methylthiotransferase), all of which may also contribute to morphological differentiation.

Cluster 19305 contains proteins annotated as integration host factors (Table S8), which are involved in site-specific recombination in Gram-negative bacteria. The syntenic region containing these genes also includes genes associated with Biosynthesis of secondary metabolites (01110), Biosynthesis of cofactors (01240), and Pyrimidine metabolism (00240). Two additional clusters—Cluster 22288 and Cluster HNH endonuclease—include proteins annotated as HNH endonucleases, which may participate in processes related to exogenous DNA integration.

Other notable clusters include Cluster 15270, which contains a DEAD/DEAH box helicase; Cluster 19368, which encodes an RNA methyltransferase; Cluster 16495, which includes a bacterial proteasome activator family protein; and Cluster 16706, which contains a cupin domain-containing protein (Table S8).

Cupins are a large and evolutionarily conserved superfamily of proteins characterized by the presence of a metal-binding cupin domain, which commonly coordinates metals such as iron and nickel [74,75]. These proteins are widely distributed across all domains of life, being found in humans, plants, and microorganisms [74,75]. In *Streptomyces*, certain cupins play key roles in regulating primary metabolism, antibiotic biosynthesis, and cellular differentiation [48,49,76]. For example, PirinA from *S. ambofaciens* is a cupin that functions as an enzymatic inhibitor of the AcdB enzyme, thereby linking primary metabolism to the synthesis of polyketide antibiotics [49]. PirA also generates reactive oxygen species that activate the CatR regulatory circuit, which is responsible for radical detoxification [48]. In *Serratia marcescens*, Pirin controls pyruvate dehydrogenase (PDH) activity and thus regulates flux through the tricarboxylic acid (TCA) cycle [77]. Another cupin protein, RmlC-like phosphomannose isomerase (SCO3025), has been identified in *S. coelicolor* [78]. This enzyme catalyzes the reversible conversion of D-fructose-6-phosphate to D-mannose-6-phosphate during the biosynthesis of GDP-mannose, an essential intermediate in cell wall formation and the production of specific glycolipids. Notably, the *S. coelicolor* Δ*manA* mutant exhibits a *bld-like* phenotype and shows reduced production of the antibiotics actinorhodin and tripyrrole red undecylprodigiosin in liquid culture [78].

Two clusters, Cluster 22655 and Cluster 10730, contain adaptive proteins annotated as tetratricopeptide repeat (TPR) proteins (Table S8). The syntenic region of Cluster 22655 includes the ribosomal gene *rpsA* (encoding small subunit ribosomal protein S1) and the *coaE* gene (encoding dephospho-CoA kinase). In contrast, the syntenic region of Cluster 10730 encompasses the *rpoE* gene and the *def* gene, which encodes peptide deformylase.

As previously described, 70 clusters were identified that contain proteins encoded by genes duplicated one or more times within the *Streptomyces* genome. Among these, 40 clusters were duplicated in only a single genome within the analyzed dataset. This indicates that such duplications are exceptional rather than widespread, suggesting they may result from recent duplication events. Many of these cluster-associated proteins are transcription factors.

Among these clusters, Cluster 1455 encodes a DeoR/GlpR family DNA-binding transcription regulator and is located in a syntenic region that also includes the *cysK* gene, which encodes cysteine synthase (Table S9). Interestingly, *cysK* is likewise syntenic with Cluster 1451, whose genes encode right-handed parallel β-helix repeat-containing proteins. Cluster 13947, which encodes an OB-fold nucleic acid-binding domain-containing protein (Table S9), is located in a syntenic region involved in potassium uptake (*trkA*) and potassium regulation mediated by a two-component system of the OmpR family (*kdpD* and *kdpE*). Cluster 16511, annotated as an Lrp/AsnC family transcriptional regulator (Table S9), is particularly interesting because its genes are located in a syntenic region that includes the *moeA* gene (molybdopterin molybdotransferase) and three dehydrogenase subunit genes, *bkdB*, *bkdA2*, and *bkdA1*, encoding 2-oxoisovalerate dehydrogenase. Notably, Cluster 16519, which encodes phenylacetic acid degradation protein PaaN (Table S9), is also located in a genomic region containing *bkdA2* and *bkdA1*, suggesting potential functional or regulatory linkage. Cluster 5490, encoding a response regulator transcription factor (Table S9), includes genes located in a region containing three respiratory chain components—*nuoA*, *nuoB*, and *nuoC*—as well as *korA* and *korB*, which encode subunits of the α-oxoglutarate/2-oxoacid ferredoxin oxidoreductase complex. Finally, Cluster 25559, annotated as a FadR/GntR family transcriptional regulator, is situated in a syntenic region that includes the *recQ* helicase gene and two previously mentioned genes, *gyrB* and *rpoD* (Table S9).

Even among the clusters duplicated in two or more genomes, several encode transcription factors. Cluster 32591, annotated as a LysR family transcriptional regulator (Table S10), is located in a syntenic region that includes three genes encoding the subunits of succinate dehydrogenase (*sdhA*, *sdhB*, and *sdhC*), an enzyme essential for cellular respiration. Interestingly, duplication of this enzyme has previously been observed in *S. ambofaciens*. The genes in Cluster 32590, which encode a putative bifunctional diguanylate cyclase/phosphodiesterase (Table S10), are located in a syntenic region that also contains genes encoding subunits of succinate dehydrogenase (*sdhA* and *sdhC*). Indeed, other clusters also contain genes that are syntenic with genes involved in the electron transport chain. For example, the genes in Cluster 1479 and Cluster 1527 are located in syntenic regions containing *fixA* and *fixB*, which encode electron transfer flavoprotein subunits (Table S10).

Among the clusters duplicated within a single genome, two—Cluster 5726 and Cluster 13513—are associated with ABC transport systems (Table S10). Cluster 5726, which encodes a ROK family transcriptional regulator (Table S10), is located in a syntenic region containing genes involved in *N*-acetylglucosamine transport (*ngcE*, *ngcF*, *ngcG*) and xylose transport (*xylH*, *xylG*, *xylHF*). In contrast, Cluster 13513, which encodes a trimeric intracellular cation channel family protein (Table S10), includes genes located in a syntenic region associated with the peptide/nickel transport system (*ABC.PE.S*, *ABC.PE.P*, *ddpF*, and *ddpD*).

Other clusters in which gene duplication was observed in two genomes are associated with teichoic acid biosynthesis and with secondary metabolism. These include Cluster 14532, encoding a CDP-alcohol phosphatidyltransferase family protein (Table S10), and Cluster 14538, encoding a glycosyltransferase family 2 protein. Both are located in a syntenic region that also contains the genes *crtB* (15-cis-phytoene synthase), *tagH* (teichoic acid transport system ATP-binding protein), *tagG* (teichoic acid transport system permease protein), and *araM* (glycerol-1-phosphate dehydrogenase). Cluster 14531, which encodes a sugar phosphate nucleotidyltransferase (Table S10), is likewise located in this syntenic region; however, its duplication was observed in 76 genomes, suggesting that this duplication event occurred earlier in evolutionary history than those of Cluster 14532 and Cluster 14538.

Clusters 14373 (encoding an acyl-CoA dehydrogenase family protein) and Cluster 14374 (encoding a MaoC family dehydratase) (Table S10) are located in syntenic regions that include several enzymes involved in intermediary metabolism, which generate precursors for secondary metabolites and link primary metabolism to antibiotic biosynthesis. Notably, these regions contain *ecm* (ethylmalonyl-CoA mutase) and *ccrA* (crotonyl-CoA reductase). In addition, three other genes are present in the same syntenic regions: *pssA* (CDP-diacylglycerol–serine O-phosphatidyltransferase), *citE* (citrate lyase subunit beta / citryl-CoA lyase), and *psd* (phosphatidylserine decarboxylase) (Table S10).

### 3.11. Results Validation and Limitations of the Genome-Wide Synteny Approach: Pirin-like Gene in Streptomyces

The approach used in this study was based on the principle that synteny can be used to infer the function of proteins of unknown function by analyzing their genomic proximity to genes with known functions. This strategy has been successfully applied in *S. ambofaciens* to deduce the functions of two proteins, AcdB and PirA. As previously reported, PirA acts as an inhibitor of AcdB, which catalyzes the first step of β-oxidation.

Interestingly, two conserved clusters included proteins whose genes were located in syntenic regions containing a *pirin-like* gene. Specifically, Cluster 16471 contained proteins annotated as SseB (33PZR) family proteins, while Cluster 16473 included proteins annotated as acyl-CoA dehydrogenases, located in a syntenic region with a gene annotated as PIR quercetin 2,3-dioxygenase (K06911). Notably, the gene encoding acyl-CoA dehydrogenase corresponds to *acdB* (COG1960), and, as reported in previous studies, the *sseB* and *acdB* genes co-occur and are located in syntenic regions not only within *Streptomyces* genomes but also in several other actinomycetes.

**Figure 6:**
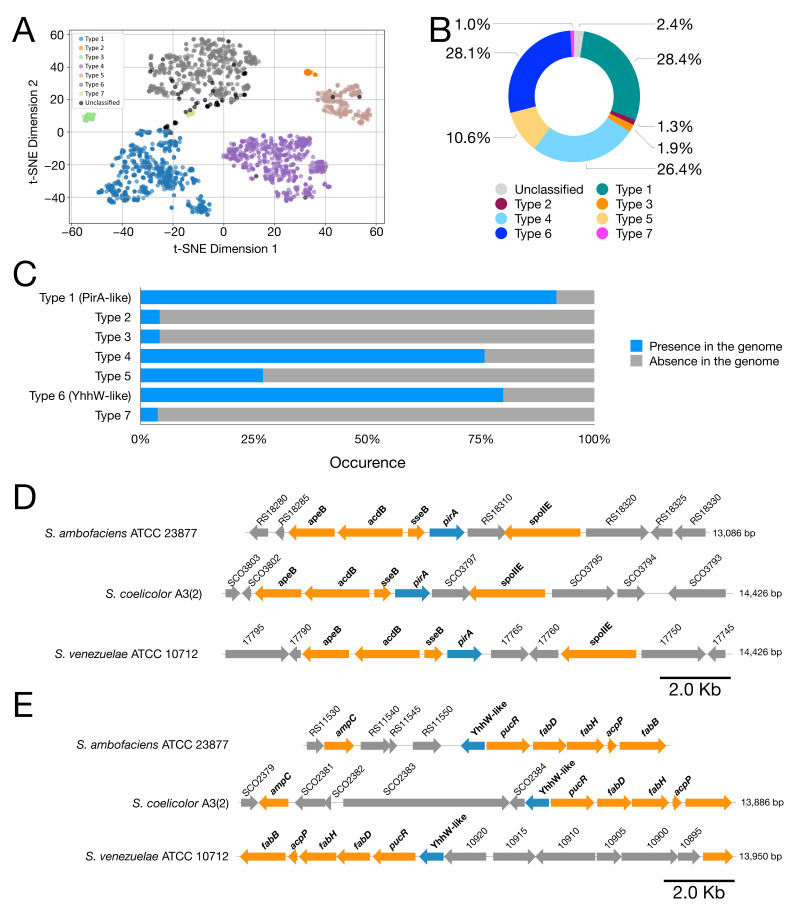
Validation of the approach using the case of the *pirA* protein of *S. ambofaciens*. (**A**) Scatter plot of *pirin-like* proteins from streptomycetes classified using the ESM-2 model. (**B**) Relative abundance of the different types of *pirin-like* proteins identified with respect to the total. (**C**) Occurrence of each type of *pirin-like* protein across the genomic dataset. (**D**,**E**) Genomic maps of regions containing the *pirA* gene (**D**) and the *yhhW-like* gene (**E**) in three streptomycetes—*S. ambofaciens* ATCC 23877, *S. coelicolor* A3(2), and *S. venezuelae* ATCC 10712.

However, to highlight potential limitations of this approach and to further validate it, an analysis of *pirin-like* sequences was performed using the deep learning protein language model ESM (Evolutionary Scale Modeling). *Pirin-like* sequences were extracted from 929 genomes, and the resulting embedding matrix was visualized using t-SNE (Figure 6A).

This system classified the *pirin-like* proteins into seven distinct types based on the embedding matrix. However, the KAAS system annotates proteins belonging to these different groups under the same code (K06911). As shown in Figure 6B, these proteins actually cluster into separate groups according to the classification obtained with the ESM model: 666 proteins were classified as Type 1, 30 as Type 2, 44 as Type 3, 617 as Type 4, 248 as Type 5, 657 as Type 6, and 23 as Type 7. Thus, the most widespread *pirin-like* proteins are those of Type 1 and Type 6. Notably, the *pirin-like* proteins whose genes are syntenic with the genes included in Cluster 16471 and Cluster 16473 belong to Type 1 in 99.5% of cases, while the remaining 0.5% were not classified by the ESM–t-SNE method.

These data confirm that the ALFATClust clustering method and KAAS annotation produce consistent results. Specifically, the genes grouped within each cluster—validated by COBALT [57]—are homologous and exhibit a high degree of similarity, while the synteny-based selection method applied to KAAS-annotated proteins allows, at least in the case of *pirin-like* genes, the selection of genes belonging to the same subtype.

Analysis of the conservation of all *pirin-like* genes within the *Streptomyces* genomes revealed that Type 1 genes—corresponding to *pirA* from *S. ambofaciens*—and Type 6 proteins are both highly conserved (Figure 6C) and represent the most abundant groups in terms of non-redundant sequences. The Type 6 pirin-like (YhhW-like) proteins are homologous to YhhW, a protein originally characterized in *Escherichia coli*.

In *S. ambofaciens*, as in many of the analyzed genomes, *pirin-like* proteins are present in multiple copies. For example, in *S. ambofaciens*, in addition to *pirA* (WP_053134172.1, Type 1) and *yhhW*-like (WP_053127613.1, Type 6) proteins, two additional *pirin-like* proteins are present: WP_053140539.1 (Type 4) and WP_053127613.1 (Type 5). Similarly, *S. coelicolor* harbors several genes encoding *pirin-like* proteins, including a *pirA*-like gene (WP_003975143.1), a *yhhW*-like gene (WP_011028320.1), one Type 4 gene (WP_011027413.1), and two Type 5 genes (WP_011031864.1 and WP_011027140.1). Interestingly, the approach employed in this study identified only genes encoding *pirA*-like proteins.

Using a targeted approach, the syntenic regions containing *pirA*-like and *YhhW*-like genes from *S. ambofaciens* ATCC 23877, *S. coelicolor* A3(2), and *Streptomyces venezuelae* ATCC 10712 were compared (Figure 6D,E). As previously reported, the syntenic region encompassing *pirA* also includes several additional conserved genes besides *acdB* and *sseB*—namely *apeB* (COG1362) and *spoIIE* (COG2208) (Figure 6D). Notably, these proteins (WP_053134165.1 and WP_053134178.1) were not annotated by the KAAS tool and, consequently, were not reported as syntenic with *sseB* and *acdB*, as occurred for the annotated *pirA* gene.

Both ApeB and SpoIIE were present in many of the analyzed genomes and were included in the initial list of clusters. However, when the clusters were filtered using the ≥75% conservation cutoff, those containing these proteins were excluded from further analyses. This finding suggests that the method employed is sufficiently sensitive, as it successfully identified the *pirA*-syntenic region. At the same time, the identified regions warrant more specific examination to detect additional, moderately conserved proteins that may have been excluded by the applied threshold.

The genomic region containing the *yhhW*-like gene was not detected by this analysis. However, manual inspection of this region in the genomes of the three examined *Streptomyces* species—*S. ambofaciens* ATCC 23877, *S. coelicolor* A3(2), and *S. venezuelae* ATCC 10712—revealed a syntenic association with genes encoding subunits of the fatty acid synthase (FAS) complex. Specifically, the *fabD*, *fabH*, *acpP*, and *fabB* genes, together with a *pucR*-like regulator (involved in fatty acid biosynthesis) and the *ampC* gene (associated with antibiotic resistance), are located in a syntenic region that also includes the *yhhW*-like gene, as shown in Figure 6E.

Both the *yhhW*-like proteins and the *fabD*, *fabH*, *acpP*, and *fabB* genes are annotated in the KAAS database, whereas the *ampC* and *pucR*-like genes are not. Notably, Cluster 20511 includes the *S. ambofaciens* protein WP_053130181.1, which was identified in the list of conserved clusters and supplementary tables. This gene was annotated as a CdaR family transcriptional regulator (COG2508) and is located in a syntenic region encompassing the previously mentioned *fabD*, *fabH*, *acpP*, and *fabB* genes. Conversely, the *ampC* gene was filtered out according to the established cutoff (>75% genome occurrence). As highlighted in Figure 6E, in some cases—such as in *S. venezuelae* ATCC 10712—*ampC* is positioned farther from the immediate genetic neighborhood considered. This is consistent with the applied synteny method, which included only the five upstream and five downstream genes relative to each cluster gene.

Finally, the genomic regions containing the two *pirin-like* genes encoding WP_053140539.1 (Type 4) and WP_053127613.1 (Type 5) were also manually analyzed. In this case, these genes were found to be less conserved than the others and located in genomic regions with a low degree of synteny.

### 3.12. Conservation and Synteny of Cluster in Other Actinomycetota

**Figure 7:**
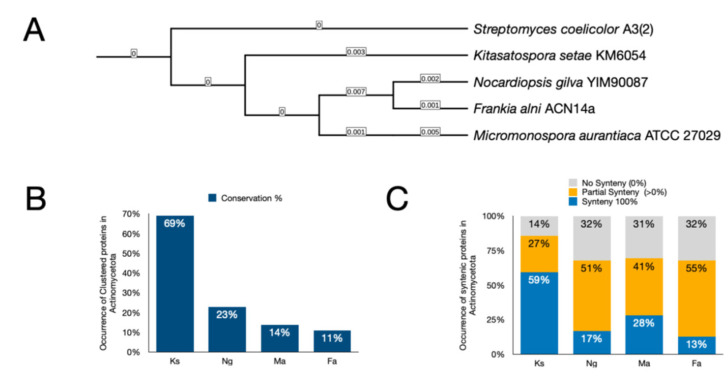
Conservation and synteny of Clusters in other Actinomycetota. (**A**) Phylogenetic tree showing the distance between S. coelicolor A3(2), *K. setae* KM6054, *N. gilva* YIM90087, *F. alni* ACN14a, and *M. aurantiaca* ATCC 27029. (**B**,**C**) Conservation (**B**) and synteny (**C**) of the 284 clusters in the four species included in this analysis. KS = *Kitasatospora setae* KM6054, Ng = *Nocardiopsis gilva* YIM90087, Fa = *Frankia alni* ACN14a; Ma *= Micromonospora aurantiaca* ATCC 27029.

To further validate and refine the results, synteny analysis was extended to four representatives of the phylum Actinomycetota: *Kitasatospora setae* KM6054, *Nocardiopsis gilva* YIM90087, *Frankia alni* ACN14a, and *Micromonospora aurantiaca* ATCC 27029 (Tables S13 and S14). Among these, *K. setae* is phylogenetically closest to streptomycetes, sharing the same order and family (Kitasatosporales; Streptomycetaceae) (Figure 7A). This phylogenetic proximity was corroborated by the degree of cluster conservation: approximately 70% of the protein clusters identified in streptomycetes were recovered in *K. setae*, a proportion that declined more steeply than in the other actinomycetes examined (Figure 7B). Synteny with KAAS-annotated genes was subsequently assessed for each cluster (Figure 7C), revealing that only 14 of the 284 clusters were conserved across all strains and harbored genes homologous to those previously identified in the *Streptomyces* database (Table 3). Among these, Cluster 3—which, as discussed above, encompasses *bldC* and several syntenic genes including *vhd* (K00271), encoding valine dehydrogenase, an enzyme implicated in metabolic differentiation in *Streptomyces*—was among the conserved clusters.

Among the conserved clusters (Table 3), several harbor genes of notable functional relevance. Cluster 41 and Cluster 91 encode a ferredoxin and the iron–sulfur cluster insertion protein ErpA, respectively, both implicated in electron transfer and Fe-S cofactor biogenesis. Cluster 9408 contains a DUF4177 domain-containing protein in synteny with a CRP/FNR family transcriptional regulator (cyclic AMP receptor protein), an uncharacterized protein, and a WhiB family redox-sensing transcriptional regulator (WhiB1/2/3/4), suggesting a potential role in redox-responsive gene regulation. Cluster 10489 encodes a DUF3117 domain-containing protein and shows near-complete sequence conservation (929/929 and 930/930 residues, 100% identity), with syntenic genes including *tatB* (sec-independent protein translocase), *folP* (dihydropteroate synthase), *rpoE* (RNA polymerase sigma-70 ECF subfamily factor), and *paaG* (2-(1,2-epoxy-1,2-dihydrophenyl)acetyl-CoA isomerase), among others. Cluster 13676 encodes a DUF2469 domain-containing protein in synteny with genes involved in DNA processing (*smf*), signal peptidase I (*lepB*), flagellar sigma factor (*fliA/whiG*), and a magnesium chelatase family protein (*comM*), pointing to possible roles in cell signalling and envelope-associated processes.

Overall, 10 of the 14 identified clusters map to syntenic regions consistent with those previously characterized in *Streptomyces* and therefore represent a high-stringency gene set with potential involvement in fundamental biological processes.

## 4. Discussion

Members of the genus *Streptomyces* are widespread across diverse environments and exhibit substantial genetic diversity, influenced by both geographical factors, such as regional diversification, primarily driven by dispersal limitation and genetic drift [79], and species-specific lifestyles, such as free-living or host-associated [80,81]. In addition, mechanisms such as horizontal gene transfer (HGT), which have been widely observed within the *Streptomyces* genus [82,83], contribute to genomic diversification, thereby shaping the evolutionary trajectories of bacterial species.

The present analysis included a total of 929 sequenced genomes belonging to the *Streptomyces* genus. This extensive dataset captures a high degree of genetic diversity, thereby facilitating the identification of genes that are conserved across species.

Indeed, the observed amino acid composition and its variance across *Streptomyces* genomes reflect the well-established correlation between genomic GC content and proteome composition that characterizes high-GC Actinobacteria [84]. In organisms with GC-rich genomes, codons for alanine (GCN), arginine (CGN, AGR), glycine (GGN), and proline (CCN) are disproportionately represented, as these amino acids are encoded predominantly by GC-rich codons. Accordingly, the high variance observed for alanine, arginine, glycine, and proline across the analyzed genomes is consistent with differences in GC content among *Streptomyces* species, where even moderate variation in genomic GC% can translate into measurable shifts in amino acid frequencies at the proteome level. This phenomenon has been extensively documented in Actinobacteria and represents a genome-wide compositional bias rather than a purely functional selection [84,85].

Conversely, amino acids encoded predominantly by AT-rich codons—such as lysine (AAA/AAG), asparagine (AAT/AAC), isoleucine (ATT/ATC/ATA), and tyrosine (TAT/TAC)—tend to be underrepresented in high-GC proteomes, which is reflected here in their consistently low abundance and reduced variance across genomes. The particularly low and invariant levels of tryptophan, cysteine, and methionine suggest that, beyond GC pressure, metabolic cost and functional constraint act as additional selective forces keeping these residues scarce across all analyzed *Streptomyces* proteomes [86].

Finally, the high variability of some amino acid residues, such as leucine and arginine, could be the result of species-specific evolution of *Streptomyces*, which could concern the repertoire of transmembrane helices for leucine [87] or the DNA-binding proteins and regulators because of the key role of arginine in electrostatic interactions with nucleic acids [88].

The amino acid compositional patterns observed in this dataset are best interpreted as the combined outcome of GC-driven mutational bias, metabolic economy constraints, and the distinctive proteome architecture of *Streptomyces*.

The genomic diversity in *Streptomyces* is evident from the data summarized in Table 1 and Figure 1. The maximum and minimum numbers of protein sequences per genome, the variation in length, and the variation in the amino acid composition (%) across the dataset highlight the diversity represented in the initial protein database used in this study.

This genetic diversity is crucial because it enables the identification of conserved genes likely subject to vertical transmission and characterized by relatively low sequence variability [89]. These include genes encoding essential components such as ribosomal proteins (*rpl1, rpl5, rpl6, rpl16, rpl18, rpl19, rps1, rps3, rps8, rps11, rps12,* and *rps13*), RNA polymerase subunits (*rpoA, rpoB,* and *rpoD*), and proteins essential for prokaryotic DNA repair mechanisms (*uvrB, uvrC, MutL,* and *RecA*), among others. Additionally, the *spoT* gene [90], which plays a central role in the stringent response, was also identified among these conserved, vertically transmitted genes [89]. Most genes of this type are well characterized and thus consistently annotated across major databases. In contrast, this study identified and analyzed several genes not annotated in the KEGG GENES database. Within the *Streptomyces* genus, 330 such genes were detected and grouped into corresponding conserved clusters. In addition, genes encoding identical proteins were also identified (Table 2). Many of these genes do not belong to the set of known vertically transmitted genes that are universally conserved among Bacteria, Archaea, and Eukaryota.  

Given the high conservation of these genes, both those encoding identical proteins and those grouped into highly homologous clusters, it is reasonable to hypothesize that the corresponding proteins participate in fundamental biological processes that remain largely unknown or poorly characterized. Among these 330 clusters, most occur as single copies per genome; however, several clusters contain genes present in multiple copies within a single genome. These duplications are not uniformly distributed across species, suggesting that such genes could serve as evolutionary markers for the *Streptomyces* genus—although further data will be required to confirm this.

Analysis of the location of conserved syntenic regions in *Streptomyces* highlights a clear contrast between the evolutionary behavior of the core and accessory genomes, as expected [91,92]. In fact, most of the 284 identified syntenic clusters are located in the “core” genomic region (Figure 4). The persistence of large syntenic blocks across phylogenetically distant strains indicates that a substantial fraction of the core genome is subject to strong constraints not only at the sequence level but also in terms of gene order and local genomic context. This structural conservation is consistent with the presence of functionally integrated modules in the “core” genome.

Interestingly, clusters containing proteins detected only once across the analyzed genomes (single-copy) show a distribution consistent with the interpretation that the core genome represents the ancestral backbone of the *Streptomyces* chromosome and is therefore enriched in conserved genes (Figure S1A, Table S8). In contrast, proteins occurring in two or more copies exhibit a more uniform distribution, including within the chromosomal arms (Figure S2A, Tables S9 and S10). This pattern suggests that duplication, recombination, or—less likely—acquisition from closely related species may underlie their broader distribution. Overall, the presence of paralogous genes in the arms appears to contribute to the pronounced genomic plasticity characteristic of *Streptomyces*.

Moreover, streptomycetes exhibit distinctive biological features, including a complex life cycle, morphological differentiation, and the formation of multicellular structures that eventually develop into unicellular spores. These characteristics—together with the high GC content typical of their genomes—may have contributed to the evolutionary diversification of streptomycetes and, more broadly, of actinomycetes from other Gram-positive bacteria. Consequently, the genes identified in this study represent promising candidates for future investigations into the genetic and functional bases of these differentiation processes. Another factor of relevance is the extraordinary metabolic versatility of streptomycetes, particularly their capacity for secondary metabolism, which underlies much of their ecological and biotechnological importance.

Many of the identified genes are directly associated with RNA polymerase (RNAP) function or, more broadly, with the process of transcriptional regulation. For instance, two of the identified clusters (clusters 4 and 12) encode the RbpA protein, a transcription factor that interacts with RNAP to stimulate transcription initiation from core promoters, but not from alternative promoters [93]. Interestingly, RbpA has been shown to indirectly enhance rifampicin tolerance in *Mycobacterium* [93]. Additionally, clusters 2989 and 9612 show similarity to SigE, while other clusters contain genes that are syntenic with *rpoE*-like genes, further supporting their role in transcriptional control. Among the identical proteins identified, the CarD factor was detected. Recent studies have demonstrated that CarD plays a critical role in modulating the stability of the RNAP–promoter complex, specifically by stabilizing the interaction between RNAP and the −10 promoter element, thereby influencing transcription initiation efficiency [94]. Indeed, substantial modifications to the transcriptional machinery can affect secondary metabolism in both *Streptomyces* and other rare actinomycetes, altering the expression of biosynthetic gene clusters and, consequently, the production of secondary metabolites. For example, some rare actinomycetes carry a second copy of the *rpoB* gene, referred to as *rpoB2*, which harbors mutations conferring rifampicin resistance. When expressed heterologously, *rpoB2* or *rpoB*(R) has been shown to enhance the biosynthesis of secondary metabolites, highlighting its role of transcription in modulating metabolic output [50,51,95,96].

Consistent with previous analyses, several genes involved in morphological differentiation, such as *bldC* and *bldD*, were identified as highly conserved [97]. BldD is a master regulator that coordinates morphological differentiation and simultaneously activates antibiotic biosynthesis; its activity is negatively regulated by the second messenger cyclic di-GMP [98,99]. BldC, a member of the MerR family of transcriptional regulators, also governs morphological differentiation. Deletion of *bldC* has been shown to delay mycelial development underscoring its essential role in developmental timing [68,100,101,102]. BldC and BldD are already well-characterized despite lacking annotation in the system used here. Nevertheless, their identification validates the effectiveness of the approach for detecting key functional genes. Importantly, several additional genes uncovered in this analysis may play roles in morphological differentiation, as they are located in conserved genomic regions syntenic with known *whi* genes.

Specifically, the genes encoding the proteins of Cluster 1 (response regulator transcription factor) are syntenic with *whiB*. Similarly, the genes of Cluster 5685 and 13676 (encoding a TetR/AcrR family transcriptional regulator and a DUF2469 domain-containing protein, respectively) are syntenic with *whiG*. The genes of Clusters 9405, 9408, 10743, and 15921 (encoding an ArsA-related P-loop ATPase, a DUF4177 domain-containing protein, a diacylglycerol kinase family protein, and a LysR family transcriptional regulator) are syntenic with genes annotated as *whiB1_2_3_4*. Additionally, the genes of Clusters 10604, 10635, and 54472 (encoding a dipeptidase, an M48 family metallopeptidase, and an ABC1 kinase family protein) are syntenic with *whiB7*, and those of Cluster 19823 (encoding a uridine diphosphate–N-acetylglucosamine-binding protein, YvcK) are syntenic with *whiA*. The *whiA, whiB, whiG*, and related *whiB*-like genes are well established as essential regulators of morphological development in *Streptomyces* [29,30,103,104,105,106,107]. Therefore, the conserved genes identified in proximity to these loci may participate in the same developmental and differentiation processes, representing promising targets for future functional characterization.

Among the other known genes identified, Cluster 6620, which includes the two-component system response regulator AfsQ1 [108], and Cluster 9608, corresponding to the two-component system response regulator CseB [109], were detected. However, as described in Section 3, many of these regulatory systems are syntenic with other uncharacterized genes encoding proteins included in the newly identified clusters, such as Cluster 9720, suggesting potential regulatory or functional associations between known and previously unannotated genes.

Furthermore, numerous metabolic genes were identified, including those involved in fatty acid metabolism, which is known to be closely interconnected with antibiotic biosynthetic pathways [48,49]. Interestingly, several metalloproteins were also detected among the conserved clusters, including peptidases and other enzyme classes potentially linked to secondary metabolism. In addition, a cupin superfamily domain-containing protein was identified. Cupins are highly conserved throughout evolution and form a large and functionally diverse superfamily, some members of which play critical roles in antibiotic biosynthesis and related metabolic processes [74,75,76,78].

Taken together, the results presented here demonstrate the effectiveness of synteny-based approaches in expanding the functional annotation of *Streptomyces* genomes beyond the limits imposed by conventional homology searches. Of the 330 conserved clusters initially identified, syntenic gene associations could be established for 284, several of which included proteins already recognized as biologically significant—such as AcdB, BldC, and RbpA—thereby validating the robustness of the approach. Notably, the application of synteny analysis enabled the reannotation of previously unannotated genes in *S. coelicolor*, corresponding to an overall expansion of genome annotation by 5%. Beyond these quantitative gains, the curated list of conserved proteins of unknown function presented in this study constitutes a prioritized resource for the scientific community, providing a high-stringency framework to guide future experimental investigations into the molecular determinants of development and secondary metabolism in *Streptomyces*. It should be noted, however, that syntenic co-localization represents a correlative line of evidence rather than functional proof, and the associations reported here will require experimental validation to establish causal relationships. Furthermore, extending phylogenetic analyses to a broader range of actinomycetes and related taxa will be important to assess the generalizability of these findings and to consolidate the annotation drawn from conserved synteny.

## 5. Conclusions

This analysis identified 330 protein-coding genes, most of which were previously uncharacterized. In total, 284 of these genes are conserved across *Streptomyces* and are located in genomic regions containing other conserved KAAS-annotated genes. The approach, which integrates genome-wide synteny and gene conservation, is an innovative hypothesis-generating framework and enables the identification of proteins involved in key molecular and biological processes in *Streptomyces*. Many of the identified genes are situated in syntenic regions containing genes associated with morphological development or enzymes that may influence bacterial metabolism. Others may affect transcription and RNA polymerase (RNAP) activity, which are known to play crucial roles in morphological differentiation and secondary metabolite biosynthesis. Although synteny analysis alone cannot yield definitive functional predictions for these proteins, it nonetheless provides a foundation for future studies aimed at experimentally elucidating the roles of these genes in *Streptomyces* development and secondary metabolism.

## Figures and Tables

**Table 1:** Overview of dataset features.

Features	n° of Sequences or (n° Protein Length)	Assembly	Species
Max n° of sequences	11,014	GCF_036227505.1	*Streptomyces* sp. NBC_01450
Min n° of sequences	4196	GCF_023614235.1	*Streptomyces* sp. MRC013
Mean n° of sequences	7469.85		
Max protein length	30019	GCF_036248255.1	*Streptomyces* sp. NBC_01717
Min protein length	14	GCF_036013605.1	*S. europaeiscabiei* NBC_00494
		GCF_036013765.1	*S. europaeiscabiei* NBC_00480
		GCF_041051895.1	*Streptomyces* sp. R08

**Table 2:** Non-redundant (identical) proteins identified in *Streptomyces* pan-proteome.

Protein ID	Multispecies Annotation	% of Genomes	Description
WP_003948845.1	Actinomycetes	99.89% (927/929)	30S ribosomal protein bS22
WP_003948568.1	Actinomycetes	98.28% (912/929)	response regulator transcription factor
WP_003948644.1	Actinomycetes	92.68% (860/929)	30S ribosomal protein S10
WP_003949541.1	Actinomycetes	91.07% (845/929)	developmental transcriptional regulator BldC
WP_003948652.1	Terrabacteria group	84.28% (782/929)	30S ribosomal protein S12
WP_003966491.1	Actinomycetes	72.98% (678 */929)	DUF3117 domain-containing protein
WP_003953493.1	Actinomycetes	68.78% (638/929)	CarD family transcriptional regulator
WP_003948630.1	Actinomycetes	58.56% (544/929)	type Z 30S ribosomal protein S14
WP_003956432.1	Actinomycetes	55.65% (517/929)	30S ribosomal protein S11
WP_003956441.1	Actinomycetes	52.42% (487/929)	50S ribosomal protein L36
WP_003959770.1	Actinomycetes	52.31% (486/929)	SsgA family sporulation/cell division regulator

* *S. coelicolor* (assembly GCF_000203835.1) DUF3117 with extended N-terminus.

**Table 3:** Conserved clusters in the other actinomycetes analyzed and co-occurrence with syntenic genes identified in the analysis of streptomycete genomes.

Cluster	Fa	Ks	Ma	Ng	Expected_K_Numbers
Cluster 3	75.0	100.0	75.0	50.0	K00271 ^Fa^^,Ma,Ks^ K00764 ^Fa,Ma,Ng,Ks^ K01933 ^Fa,Ma,Ng,Ks^ K03578 ^Ks^
Cluster 41	50.0	100.0	100.0	50.0	K01439 ^Fa,Ma,Ng,Ks^ K22476 ^Ma,Ks^
Cluster 91	100.0	100.0	100.0	75.0	K00856 ^Fa,Ma,Ks,Ng^ K02275 ^Fa,Ma,Ks,Ng^ K03517 ^Fa,Ma,Ks^ K04487 ^Fa,Ma,Ks,Ng^
Cluster 9408	100.0	100.0	66.7	100.0	K09117 ^Fa,Ma,Ks,Ng^ K10914 ^Fa,Ks,Ng^ K18955 ^Fa,Ma,Ks,Ng^
Cluster 9693	50.0	100.0	100.0	50.0	K01011 ^Fa,Ma,Ks,Ng^ K03711 ^Ma,Ks^
Cluster 10489	66.7	83.3	66.7	50.0	K00796 ^Fa,Ma,Ks^ K01246 ^Ma,Ks^ K03088 ^Fa,Ks,Ng^ K03117 ^Fa,Ks^ K08372 ^Fa,Ma^ K15866 ^Ma,Ks,Ng^
Cluster 13676	80.0	60.0	80.0	80.0	K02405 K03100 ^Fa,Ma,Ks,Ng^ K04096 ^Fa,Ma,Ng^ K07391 ^Fa,Ma,Ks,Ng^ K07460 ^Fa,Ma,Ks,Ng^
Cluster 15448	0.0	0.0	100.0	0.0	K00012 ^Ks^
Cluster 16495	0.0	100.0	0.0	0.0	K10716 ^Ks^ K12132 ^Ks^
Cluster 19331	88.9	100.0	44.4	77.8	K01159 ^Fa,Ks,Ng^ K03072 ^Fa,Ks^ K03210 ^Ks^ K03550 ^Fa,Ks,Ng^ K03551 ^Fa,Ks,Ng^ K06215 ^Fa,Ma,Ks,Ng^ K08256 ^Fa,Ma,Ks,Ng^ K08681 ^Fa,Ma,Ks,Ng^ K22311 ^Fa,Ma,Ks,Ng^
Cluster 19775	0.0	0.0	0.0	0.0	K01628 K06889
Cluster 19814	83.3	83.3	83.3	83.3	K03297 K04488 ^Fa,Ma,Ks,Ng^ K05710 ^Fa,Ma,Ks,Ng^ K09013 ^Fa,Ma,Ks,Ng^ K09015 ^Fa,Ma,Ks,Ng^ K11717 ^Fa,Ma,Ks,Ng^
Cluster 20801	100.0	100.0	0.0	0.0	K03570 ^Fa,Ks^ K05515 ^Fa,Ks^ K05837 ^Fa,Ks^
Cluster 33898	75.0	100.0	50.0	75.0	K02346 ^Ks^ K08744 ^Fa,Ma,Ks,Ng^ K08999 ^Fa,Ma,Ks,Ng^ K16881 ^Fa,Ks,Ng^

KS = *Kitasatospora setae* KM6054, Ng = *Nocardiopsis gilva* YIM90087, Fa = *Frankia alni* ACN14a, Ma = *Micromonospora aurantiaca* ATCC 27029.

## Data Availability

The original data presented in the study are openly available in Zenodo at DOI: 10.5281/zenodo.18784887.

## Supplementary Materials

The following supporting information can be downloaded at: https://www.mdpi.com/article/10.3390/microorganisms14040791/s1, Figure S1: Clusters containing the same number of proteins were counted only once. (**A**). Observed distribution curve. (**B**,**C**). Nonlinear model fitting performed using PAST software: (**B**) increasing decay model and (**C**) Hill’s sigmoid curve; Figure S2: Distribution of genomic coordinates for clusters containing single-copy proteins (**A**) and multi-copy proteins (**B**) across the 929 genomes analyzed. Clusters that include conserved proteins and that are located in conserved genomic regions (284 of 330) were analyzed; Table S1: Accession numbers of the genomes used in the analysis; Table S2: Number of protein sequences per genome, maximum and minimum protein lengths, and overall characteristics of the dataset; Table S3: List of conserved cluster names occurring no more than once per genome; Table S4: List of conserved cluster names occurring no more than twice in a single genome; Table S5: List of conserved cluster names occurring no more than twice across two or more genomes; Table S6: List of conserved cluster names occurring no more than three times across two or more genomes; Table S7: List of conserved clusternames occurring multiple times within the same genome; Table S8: Genes syntenic to those included in clusters occurring only once per genome; Table S9: Genes syntenic to those included in clusters occurring no more than twice per genome; Table S10: Genes syntenic to those included in clusters occurring more than twice per genome; Table S11: OR and FDR values calculated on syntenic proteins annotated with the KAAS system (all KEGG numbers of the syntenic proteins were treated as a single list); Table S12: OR and FDR values calculated for syntenic proteins annotated with the KAAS system considering the clusters separately (one list for each cluster); Table S13: Genes identified using Blast in the genome of *K. setae* KM6054, *N. gilva* YIM90087, *F. alni* ACN14a, *M. aurantiaca* ATCC 27029. Coverage cutoff >70%; Table S14: Conservation of clusters identified in *Streptomyces* in other Actinomycetota (*K. setae* KM6054, *N. gilva* YIM90087, *F. alni* ACN14a, *M. aurantiaca* ATCC 27029).

## Abbreviations

The following abbreviations are used in this manuscript:

ESM-2	Evolutionary Scale Modeling 2
L-TIRs	long terminal inverted repeats
BGCs	Biosynthetic Gene Clusters
ID	protein identifier code (accession number)
R-D	Redundant Dataset
NR-D	Non-Redundant Dataset
NR-U-D	Non-Redundant Unannotated Dataset
C-NR-D	Clustered Non-Redundant Dataset
KEGG	Kyoto Encyclopedia of Genes and Genomes
KAAS	KEGG Automatic Annotation Server
COG	Cluster of Orthologous Groups
PKS	Polyketide Synthases
FAS	Fatty Acid Synthase
DUF	Domain of Unknown Function
