# Supplementary figures and images for: Functional Assessment of Orphan Proteins in the Streptomyces Pan-Proteome Through Genome-Wide Synteny Analysis
Source: Microorganisms. 2026 Mar 31;14(4):791. doi: 10.3390/microorganisms14040791 (PMC13118837; doi:10.3390/microorganisms14040791)

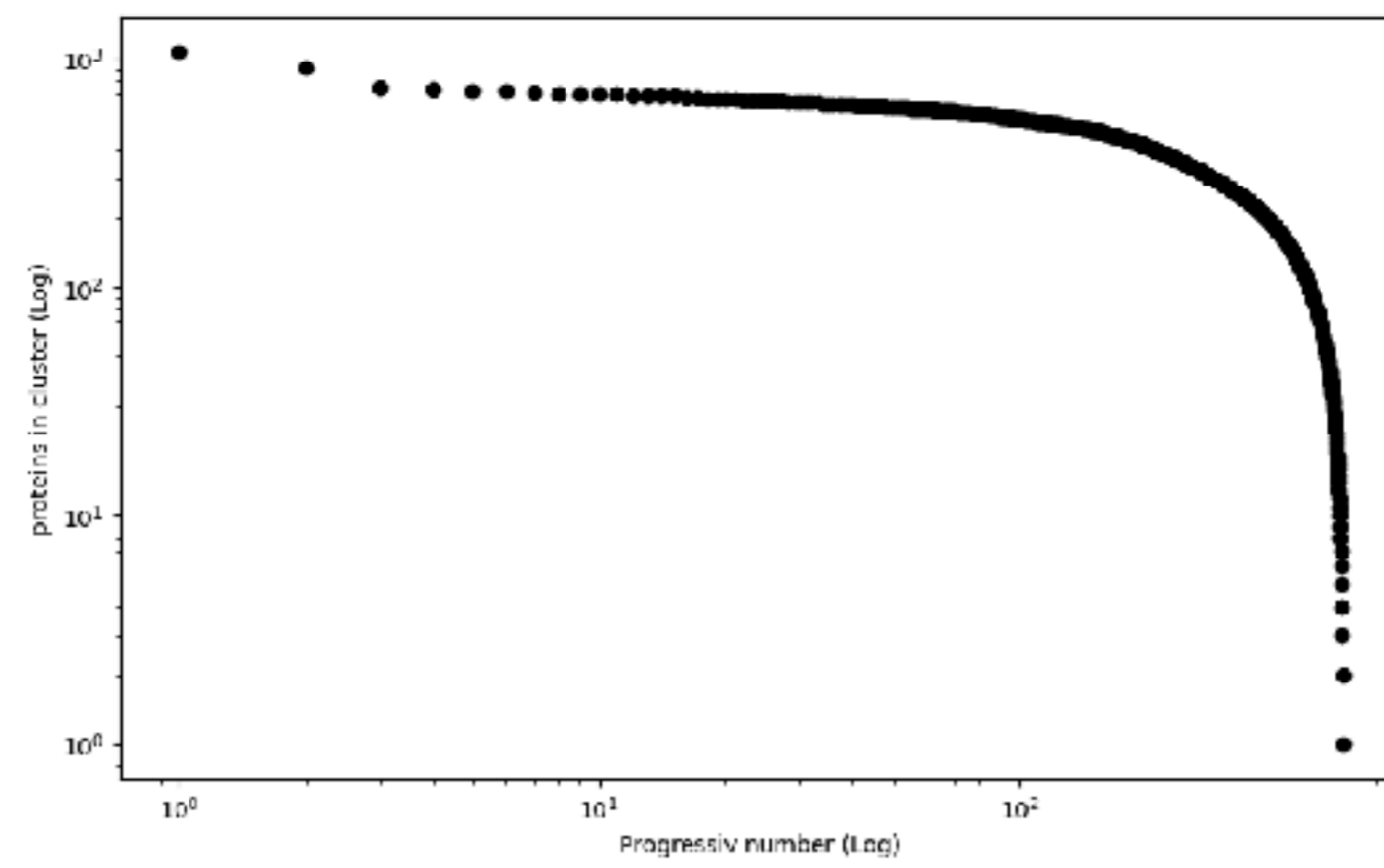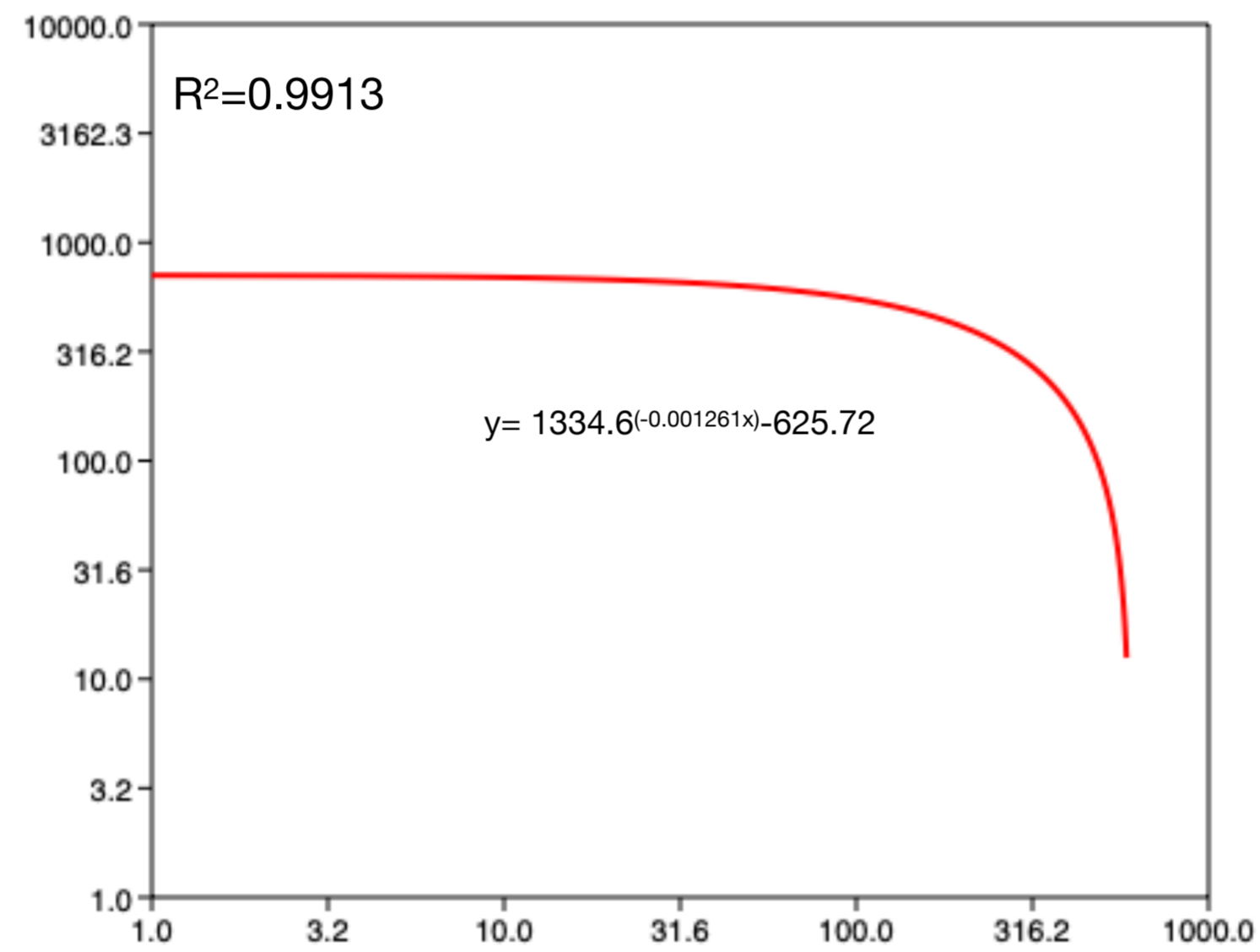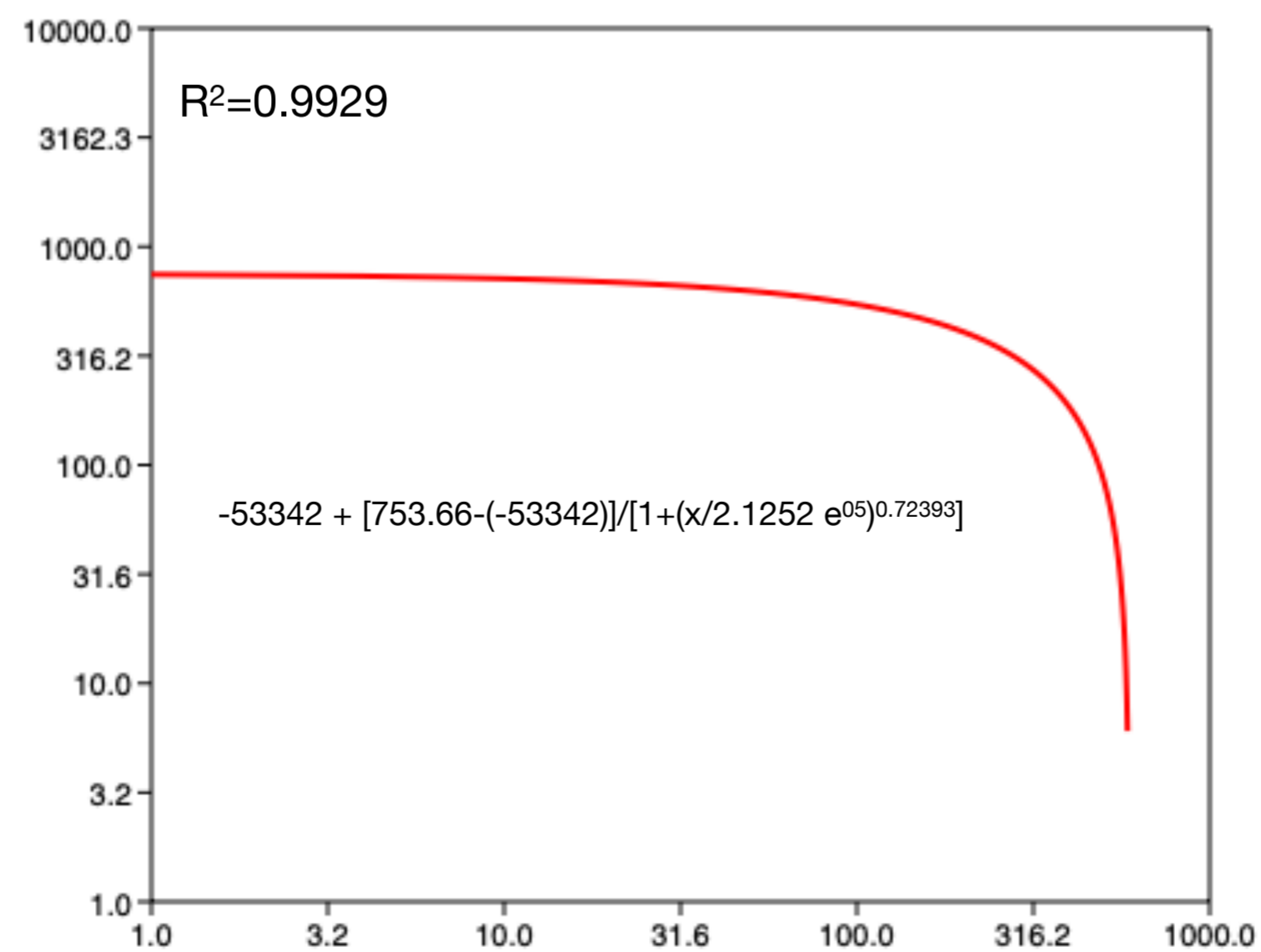

Supplement: Supplementary file 1 [file microorganisms-14-00791-s001.zip › Conserved_ORF_Streptomyces_Figure_S1.pdf]

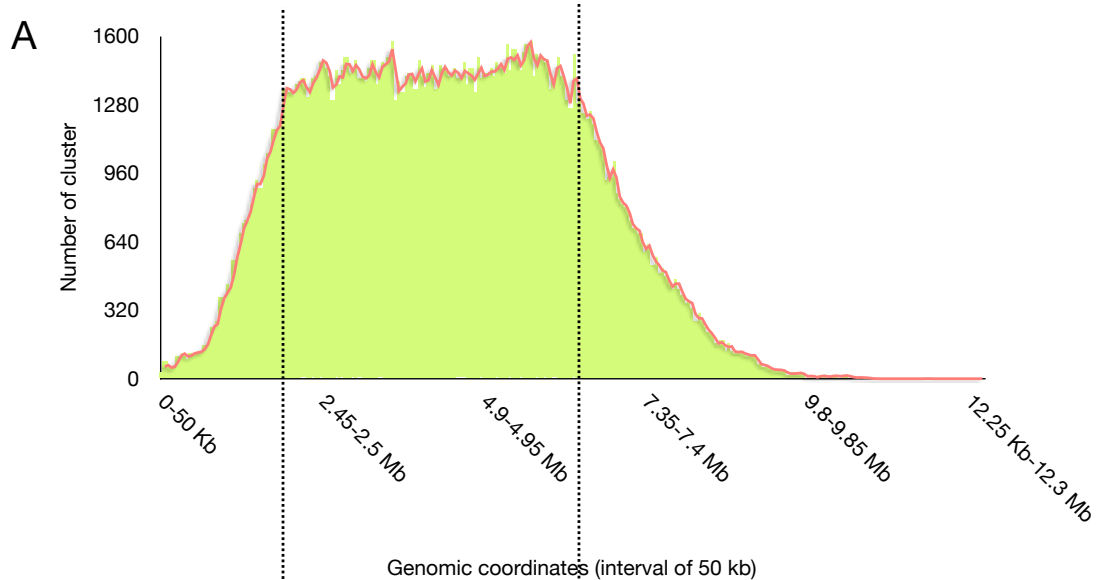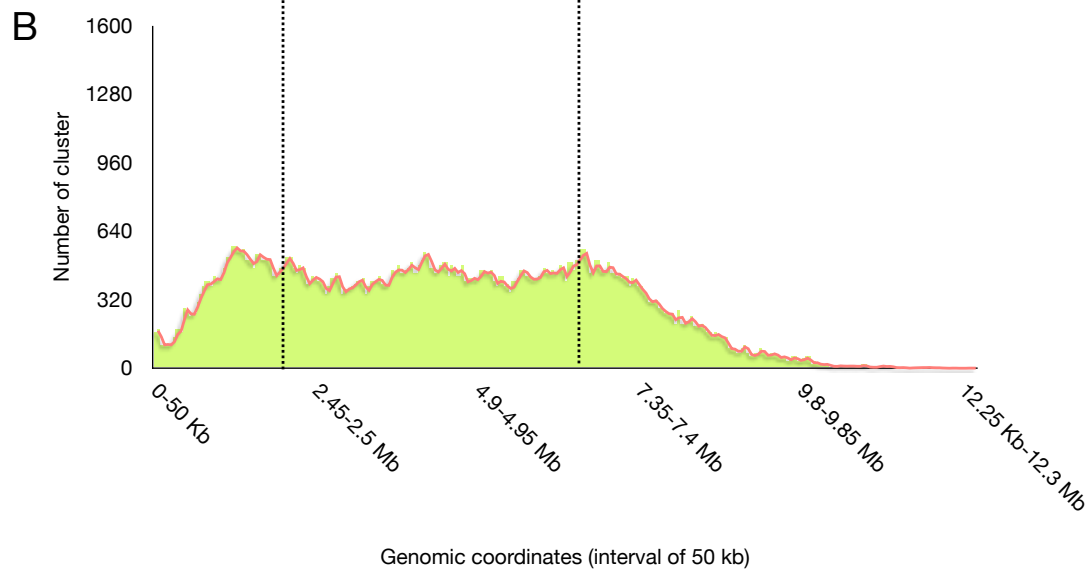

Supplement: Supplementary file 1 [file microorganisms-14-00791-s001.zip › Conserved_ORF_Streptomyces_Figure_S2.pdf]
